# Phylogenetic and structural analyses reveal Cdc2-like kinases (CLKs) as ancient regulators of thermosensitive splicing

**DOI:** 10.1016/j.jbc.2025.110979

**Published:** 2025-11-26

**Authors:** Rachel A. Ogle, Jacob K. Netherton, Benjamin R. Robinson, Florian Heyd, Mark A. Baker

**Affiliations:** 1 Faculty of Science and Faculty of Health and Medicine, University of Newcastle, Callaghan, Nova ScotiaW, Australia; 2 Institut für Chemie und Biochemie, RNA Biochemie, Freie Universität Berlin, Berlin, Germany

**Keywords:** alternative splicing (AS), pre-mRNA processing, protein structure, molecular evolution, serine/threonine protein kinase, protein phosphorylation, last eukaryotic common ancestor (LECA), Cdc2-like kinases (CLKs), LAMMER kinase, serine/arginine-rich (SR) proteins

## Abstract

The family of Cdc2-like kinases (CLKs) plays a critical role in regulating both constitutive and alternative pre-mRNA splicing. Of particular interest, CLKs exhibit unique thermosensitive properties characterized by increased activity at lower physiological temperatures. In this study, we integrate phylogenetic, protein-interaction, and structural analyses to investigate the evolutionary history and functional adaptation of CLKs across protista, fungi, plants, and metazoans. Our phylogenetic analysis, comprising 149 CLK homologs from 86 species, traces this gene back to the last eukaryotic common ancestor. The results reveal lineage-specific patterns of gene duplication and loss, including complete loss of CLKs in seven protist lineages and in microsporidian fungi. Interolog mapping identified 92 conserved CLK-interacting proteins across diverse species. In metazoans, these conserved interactors are primarily involved in complex splicing regulation, whereas in yeast they are associated with simplified RNA-processing mechanisms. Comparative structural modeling shows strong conservation of the kinase domain throughout eukaryotes, although notable divergence occurs in some Fungal and Protista lineages. Intrinsic disorder in the CLK N terminus emerges as a conserved structural property; however, sequence variability in this region modulates kinase activity and substrate specificity. Structural conservation in the activation segment, the core driver of CLK thermosensitivity, is observed across all eukaryotic kingdoms, though deviations were identified in various protist and plant lineages. Deleterious mutations often occur in this region following a duplication or preceding complete gene loss. Finally, species-specific temperature activity profiles underscore the adaptive evolution of CLKs, enabling organisms to thrive in diverse environmental conditions, including extreme temperatures.

The family of Cdc2-like kinases (CLKs) possesses dual-specificity, enabling them to phosphorylate serine/threonine, and tyrosine residues. Despite sharing structural similarities with Cdc2, CLKs have distinct functions. Throughout eukaryotes, CLK homologs are also termed LAMMER kinases due to the presence of the conserved “EHLAMMERILG” motif, and have been studied in human (1, 2), mouse (3), fruit fly (4), roundworm (5), turtle (6), alligator (6), frog (7, 8), plants (9, 10), and yeast (11, 12). The number of genes in this family increases with organism complexity, with mammals possessing a total of four paralogs (CLK1, CLK2, CLK3, and CLK4). However, limited research has explored the genetic and functional diversification of this gene family. Although some partial phylogenetic analyses of CLKs have been performed elsewhere (10, 12, 13, 14), to date, no study has demonstrated a complete eukaryotic timeline, which could offer valuable insights in predicting functional roles and diversification across species (15). Similarly, while crystal structure comparisons of human CLK1-4 have been determined (1, 2, 16, 17), there exists a gap in evaluating the structural conservation of CLK homologs among eukaryotes.

An important and well-known function of the CLK family is their role in regulating both constitutive and alternative pre-mRNA splicing through the phosphorylation of serine/arginine-rich proteins (SR proteins), which is conserved from mammals to plants (2, 10, 18, 19). Alternative splicing (AS) is a fundamental process for multicellular eukaryotes, facilitating the production of multiple protein isoforms from a single gene and contributing to their ability to adapt and diversify (20). Mutations affecting splice site recognition account for about 15% of all hereditary disease-causing mutations in humans, highlighting the importance of AS (21). Chemical inhibition of CLKs causes global changes in AS (6, 10). Hence, it is not surprising that aberrant CLK expression in humans has been extensively linked to various diseases, including cancer, muscular dystrophy, Alzheimer's, osteoarthritis, and viral replication (reviewed in detail elsewhere (1, 2, 22)). For this reason, CLKs are being rapidly established as effective targets for therapeutic intervention, highlighting the importance of understanding their functional biology.

Organisms continuously utilize AS to rapidly shift their intercellular processes in response to environmental cues. In the context of CLKs, a notable function is their ability to regulate AS in response to small changes in temperature. This newly discovered feature is especially unique, such that their activity is upregulated by just a 1 °C decrease in physiological temperature, which is in stark contrast to typical kinase thermodynamics. This phenomenon has been observed in CLK homologs across diverse eukaryotes, including human, mouse, fruit fly, turtle, alligator, plant, and thermophilic algae (6, 10, 23, 24), suggesting this mechanism is likely of ancient eukaryotic origin. This unique adaptation allows CLKs to promptly alter AS in a temperature-dependant manner by modulating SR protein phosphorylation. This function has potential links to the regulation of important biological processes, including circadian rhythms in mammals, reptilian temperature-dependent sex determination, plant thermomorphogenesis, and others (6, 10, 25). Considering that all eukaryotic organisms experience temperature fluctuations, this positions CLKs as potential regulators of an immense range of biological processes. Remarkably, the temperature profile at which CLK homologs are regulated has adapted to the specific physiological environment of each host organism. For example, in mammals, activity of CLK1/4 is typically regulated between 33 to 38 °C. However, in organisms that inhabit more extreme conditions, such as the thermophilic red algae *Cyanidioschyzon merolae*, temperature-regulation of its CLK homolog “LIK” occurs between 48 to 56 °C (23). Despite variations in their activity profile, a key aspect to their functionality is that all CLKs examined so far, exhibit increased activity below an organism’s physiological temperature.

This article aims to address knowledge gaps on the evolution and diversification of the CLK family of kinases. Our approaches include the following: (1) a phylogenetic analysis demonstrating CLK diversification and gene gain/loss, (2) networking orthologous protein-protein interactions to explore conserved functions (3) structural comparisons of their kinase domain and N termini to uncover conservation, and (4) an investigation into how their unique temperature regulatory mechanisms have evolved and diversified. Although the importance of CLKs in the context of humans is rapidly expanding due to their association with various diseases, there is a substantial body of research in other model species that could shed light on their function. Thus, this work offers insights into the functional evolution of the CLK family, serving as a resource for researchers interested in studying these kinases across eukaryotic organisms.

## Results and discussion

### Phylogenetic history of CLKs

To investigate the evolutionary origins and diversification of the CLK kinase family, we identified CLKs using NCBI’s Conserved Domains Database (CDD). Four profiles were retrieved, including cd14134 (CLK), encompassing all eukaryotes outside of vertebrates, and cd14213 (CLK1_4), cd14214 (CLK3), and cd14215 (CLK2), which were exclusive to vertebrate species. We extracted CLK sequences using the CDD annotations in UniProt and then employed HMMER to construct hidden Markov model (HMM) profiles using the CDD portion of the sequence. These profiles were subsequently used to search the NCBI RefSeq protein database to identify CLKs across eukaryotes. To bolster our analysis, we repeated this process, using the initial search results to generate new HMM profiles to be re-searched.

We manually curated our dataset to distinguish genuine genomic events (such as duplications and gene loss) from database errors, a common issue reported in previous studies (26, 27, 28, 29, 30, 31). As such, we erred on the side of caution and prioritized events that were consistent among closely related species (for details on our selection criteria, see Experimental Procedures). CLKs were identified based on their best 1 domain E-value from the HMMER search, although additional BLAST searches were required in cases where significant divergence (high E-value) had occurred. Due to challenges with sequence alignment, we only investigated these highly diverged CLKs in species where this was the sole gene. As such, it is likely that more CLK duplications have occurred during eukaryotic evolution and were subsequently followed by significant divergence/loss of function. The complete HMMER dataset with all included/excluded genes can be found in Table S1.

**Figure 1:**
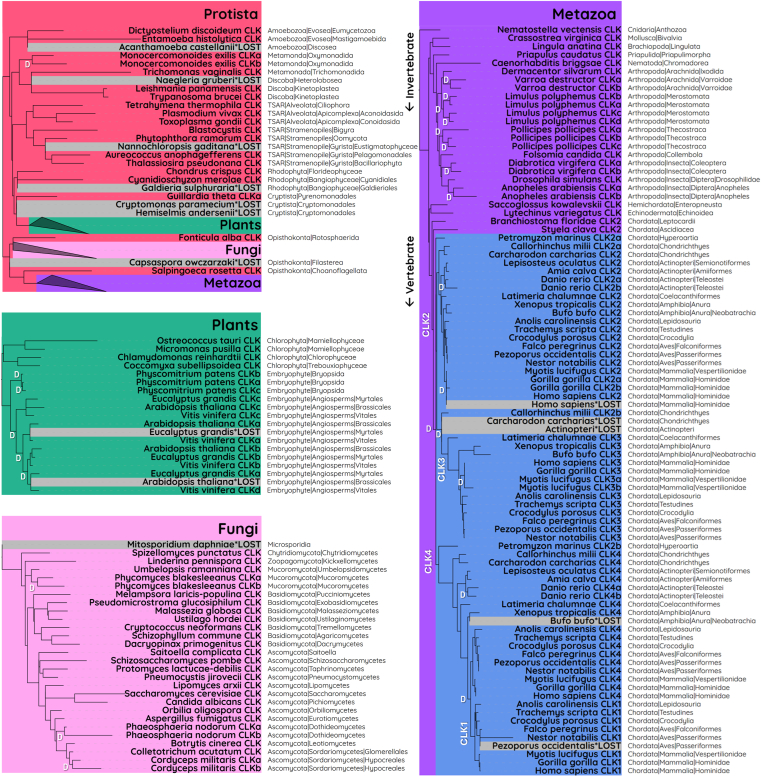
**Phylogenetic analysis of CLK proteins across eukaryotes reveals lineage-specific patterns of gene duplication and loss**. CLK proteins were identified in the NCBI RefSeq database using generated HMM profiles. The kinase domains were aligned using 3D-Coffee, and a phylogenetic tree was constructed using a species-aware maximum likelihood approach (RAxML-NG and GeneRax). A total of 149 CLK homologs from 86 eukaryotic species are shown. Branch lengths indicate evolutionary divergence. *White* "D" marks gene duplication events. CLK, Cdc2-like kinase.

For the phylogenetic analysis, species were selected to represent the majority of eukaryotic taxonomic groups and those with gain/loss events. Given the large evolutionary distances and high sequence divergence, especially among Protista, we used the structure-based T-Coffee alignment method, 3D-Coffee (32). A total of 149 CLK homologs from 86 species were used to construct a phylogenetic tree with a species-tree-aware maximum likelihood approach (Fig. 1). In nonvertebrate species, CLK genes were uniformly labeled as “CLK,” while in vertebrates, genes were named according to to the corresponding HMM profile (CLK1-4) that produced the lowest E-value. Lowercase letters (*e*.*g*., CLKa and CLKb) were added to differentiate copies, where gene duplications occurred. Although these letters were not assigned in a specific order, we maintained consistency across orthologs in closely related species where possible.

To assess whether CLKs are exclusive to eukaryotes, we screened Bacteria and Archaea RefSeq databases. No definitive evidence of CLK homologs were identified beside a short 90 amino acid sequence from *Gelidibacter salicanalis (*NCBI: WP_199603858.1). However, given that the bacterial sample was isolated from a eukaryotic copepod host (33), and the sequence shares 97% similarity to the copepod *Tigriopus californicus*, we suggest this is likely a contamination error. As such, the absence of CLKs in prokaryotes supports our findings that the ancestral CLK gene emerged during eukaryogenesis and was present in the last eukaryotic common ancestor (LECA) (34, 35).

#### Protista

Protists, a paraphyletic group of eukaryotes distinct from animals, plants, and fungi, exhibit the highest diversification of CLK proteins, as reflected by their high E-values (Table S1) and long branch lengths (Fig. 1, red). Remarkably, we identified seven independent protist lineages that appear to have lost CLK genes entirely: Heterolobosea, Discosea, Eustigmatophyceae, Pyrenomonadales, Filasterea, Cryptomonadales, and Galdieriales. Of these, only three lineages included multiple species within the database, while five were each represented by a single species. As such, incomplete database annotations may explain some apparent gene absences, and further sequencing could reveal CLKs within these species.

Within Amoebozoa, species in the Evosea clade retain a single CLK gene, whereas a species in the Discosea clade has completely lost it. In Mastigamoebida (Evosea), *Entamoeba histolytica*, along with four other *Entamoeba* species, display highly diverged CLK sequences. This suggests a functional shift and ongoing trend toward gene loss, which aligns with the complete loss observed in *Acanthamoeba castellanii* of the Discosea.

Among Metamonada, *Trichomona vaginalis* (Trichomonadida) harbors a single, highly diverged CLK gene, whereas two CLKs are observed in *Monocercomonoides exilis* (Oxymonadida), both of which are less diverged in comparison. Although *M*. *exilis* entirely lacks mitochondria, its genomic content is suggested to be closer to the LECA and less reduced than other metamonad protists like *T*. *vaginalis* (36).

In the Discoba clade, species of the class Kinetoplastea possess a single CLK gene, while three *Naegleria* species (Heterolobosea) have lost their CLK entirely. This is an intriguing case given that the *Naegleria gruberi* genome is considered to be relatively complex compared to other protists (37). Previous research on kinetoplastid parasites, including *Trypanosoma* and *Leishmania* species, highlights how CLK proteins have evolved a specialised role within the unique kinetochores of these organisms (38, 39, 40, 41). Although some studies report a recent duplication yielding two identical CLK paralogs in these organisms, our analysis suggests that this apparent duplication is likely a database error. In this case, the reported CLK duplicates within individual kinetoplastid species are completely identical, lacking any mutations, while these CLK duplicates between different kinetoplastid species display substantial divergence. This pattern indicates that these sequences are unlikely to have arisen from an ancestral duplication event within kinetoplastids; instead, they most likely represent a single gene.

Our analysis revealed that the only Haptista species represented in the NCBI RefSeq database, *Emiliania huxleyi*, lacks a detectable CLK gene. However, BLASTP searches identified four other species within this phylum each of which contains a gene homologous to the CLK family: *Chrysochromulina tobinii* (GenBank: KOO29502.1), *Diacronema lutheri* (GenBank: KAG8459592.1), *Prymnesium parvum* (GenBank: KAJ1623349.1), and *Pavlovales* sp. CCMP2436 (GenBank: KAJ1617502.1). This discrepancy may reflect a missing annotation in the *E*. *huxleyi* RefSeq genome; however, this species is also known for substantial gene content variability among environmental isolates, underscoring the dynamic nature of gene gain and loss in this lineage (42).

All examined Alveolata species (including Ciliophora and Apicomplexa) within the TSAR supergroup contain a single CLK gene. This trend also holds for Stramenopiles from the phyla Oomycota and Bigyra, as well as diatoms (Bacillariophyta) within Gyrista. However, one Gyrista species, *Aureococcus anophagefferens* (Pelagomonadales), appears to have lost CLK.

In red algae (Rhodophyta), known for extensive genome reduction and loss of splicing components (43), *Cyanidioschyzon merolae* and *Chondrus crispus* each retain a single CLK gene. In contrast, *Galdieria sulphuraria* lacks CLK entirely, despite having a more intron-rich genome and extensive splicing machinery compared to *C*. *merolae* (43). This suggests that *G*. *sulphuraria* may have evolved CLK-independent splicing mechanisms. Within Cryptista, two species from the order Cryptomonadales have lost their CLK genes, while *Guillardia theta* (Pyrenomonadales) exhibits a CLK duplication. The additional paralog, “CLKb,” was excluded from our phylogenetic tree due to its unresolved placement, although it often clustered near the TSAR lineage, this was inconsistent. Though this may reflect high sequence divergence, it is also possible this gene originated *via* horizontal gene transfer, which is known to have occurred extensively throughout the *G*. *theta* genome (44, 45).

Finally, our analysis included three protist species within the Opisthokonta clade, a group that encompasses both animals and fungi. These species, *Fonticula alba* (Rotosphaerida), *Capsaspora owczarzaki* (Filasterea), and *Salpingoeca rosetta* (Choanoflagellata), represent key evolutionary intermediates between unicellular protists and multicellular metazoans. Both *F*. *alba* and *S*. *rosetta* retain a single CLK gene, suggesting a conserved role for this kinase in these lineages. In contrast, *C*. *owczarzaki* has lost its CLK gene entirely, an unexpected finding given its complex AS regulation throughout its life cycle (46) and considerable intron density, averaging 3.8 introns per gene (47). The complete absence of CLK in this species suggests that other mechanisms may have evolved to control alternative splicing. Investigating how *C*. *owczarzaki* compensates for the loss of CLK could provide deeper insights into the core functions of CLK proteins.

#### Plants

In the Viridiplantae kingdom (Fig. 1, green), unicellular green algae (Chlorophyta) possess a single CLK gene, as observed in *Chlamydomonas reinhardtii* (Chlorophyceae), *Ostreococcus tauri*, and *Micromonas pusilla* (Mamiellophyceae), and *Coccomyxa subellipsoidea* (Trebouxiophyceae).

Following the divergence of chlorophytes, land plants (Embryophyta) experienced an expansion of the CLK gene family, shaped by independent gene duplications and losses. In the early diverging moss, *Physcomitrium patens* (Bryopsida), the presence of three CLK genes reflects two lineage-specific duplication events. Gene expansion also occurred in flowering plants (angiosperms), where *Arabidopsis thaliana* (Brassicales) and *Eucalyptus grandis* (Myrtales) each have three CLK paralogs, and *Vitis vinifera* (Vitales) has four. Interestingly, only one CLK gene pair between *A*. *thaliana* and *E*. *grandis* is truly orthologous; the others are pseudoorthologs—paralogs that mimic orthology due to lineage-specific gene loss. This suggests that the ancestral duplications, as seen retained in *V*. *vinifera*, were independently lost in the other two species.

The expansion of the CLK family in land plants coincides with the evolution of complex multicellularity and terrestrial adaptation. This is consistent with studies showing that the transition to land was driven by both whole-genome and single-gene duplications, which enabled plants to adapt to challenges such as drought, nutrient limitation, and increased UV radiation (48).

#### Fungi

Among fungi, all species in our analysis have retained at least one CLK gene except for microsporidia, which lack CLK entirely (Fig. 1, pink). We detected no CLK genes in *Mitosporidium daphniae*, alongside nine other diverse microsporidia species, a finding that mirrors the extensive genome reduction typical of these obligate intracellular parasites (49). Our results also reveal considerable sequence divergence in fungal CLKs, though to a lesser degree than those observed in protist lineages. Various independent CLK duplications were identified in diverse fungal lineages as discussed below, some of which are included in the phylogenetic analysis (Fig. 1, pink), while the remaining are listed in Table S1.

In early diverging fungal groups, both identified species within Zoopagomycota contain a single CLK gene, while among the five Chytridiomycota species, all possess a single CLK gene except for *Synchytrium microbalum*, which exhibits a duplication. Within Mucoromycota, gene duplication/retention varies: *Umbelopsis ramanniana* maintains one copy, whereas the species *Phycomyces blakesleeanus* and *Mucor velutinosus* possess a gene duplication. Although most species in this fungal lineage typically have 1 to 2 CLKs, *Lichtheimia ornata* possesses 4 CLKs, suggesting additional duplications have occurred in this species. Interestingly, the CLK duplicates among Mucoromycota are often either diverged (reflected in high E-values), or entirely absent, suggesting a trend toward pseudogenization and loss of function through mutation accumulation.

In Basidiomycota, all analyzed species were found to harbor a single CLK gene, including the *Cryptococcus*, *Ustilago*, and *Schizophyllum* genera. Similarly, most Ascomycota, such as *Saccharomyces cerevisiae*, *Schizosaccharomyces pombe*, and members of the Dipodascomycetes and Eurotiomycetes, also possess just one copy. However, six cases of duplication were identified within Ascomycota: five in Sordariomycetes and one in Dothideomycetes. Within the former, independent duplications were seen in *Ilyonectria robusta*, *Drechmeria coniospora*, and *Phialemonium atrogriseum*, even though related species within the same families lack them. Among the four Cordycipitaceae species, an ancestral duplication was identified, with phylogenetic analysis of “CLKb” in *Cordyceps militaris* showing divergence post duplication. An independent diverged CLK duplicate was identified in *Apiospora marii*, although other species in the same genus only possess one CLK. Divergence post duplication was also seen in the Dothideomycetes species, *Parastagonospora nodorum* (Phaeosphaeriaceae), with one paralog “CLKb” showing significant divergence, as indicated by its longer branch length. This pattern of divergence is common following gene duplication, as the initial redundancy permits one copy to accumulate mutations—either leading to novel functions or eventual gene loss (50, 51).

Excluding microsporidia, the consistent retention of at least one CLK gene across all fungal species implies that these kinases perform essential functions. Although genetic knockouts of the sole CLK gene in six different yeast species have produced viable organisms, many exhibit negative effects such as reduced growth and virulence (11, 52, 53, 54, 55, 56, 57). This, along with the strong gene retention observed among fungi, indicates that while immediate survival may not be compromised by CLK loss, these kinases are likely critical for long-term fitness. In contrast, the loss of CLK genes in various protist lineages and microsporidia shows that some unicellular eukaryotes have evolved alternative mechanisms to function without this gene. Investigating the functional implications of CLK loss in these organisms may offer valuable insights into the essential roles of CLK proteins in eukaryotes.

#### Invertebrate

Across metazoan lineages, every species analyzed retains at least one CLK gene, underscoring its essential role in complex organisms (Fig. 1, purple). Among early invertebrates, species in the phyla Cnidaria, Brachiopoda, Mollusca, Priapulida, and Nematoda each possess a single CLK copy, while flatworms (Platyhelminthes) exhibit a duplication event.

Independent duplications of CLK genes are frequent within arthropods. For instance, *Pollicipes pollicipes* (Thecostraca) exhibits two independent duplications. In Diptera, *Anopheles* species show a duplicated CLK gene, in contrast to other dipterans like *Drosophila*, which maintain a single copy. In the family Chrysomelidae (Coleoptera), a duplication is observed in the beetle *Diabrotica virgifera*. Among arachnids, a duplication event occurred in Mesostigmata, whereas *Dermacentor silvarum* (Ixodida) retains only a single CLK gene.

Among deuterostome invertebrates, species in Echinodermata, Hemichordata, and Chordata all harbor a single CLK gene. It is important to note that the chordate invertebrates were unexpectedly found within the vertebrate RefSeq database and were thus analyzed with the vertebrate CLK1–4 CDDs. Consequently, the sole CLK gene identified in sea squirts (Ascidiacea) and lancelets (Leptocardii) aligns best with the CLK2 CDD (cd14215) and is named accordingly, supporting the hypothesis that the CLK2 most closely resembles ancestral CLK.

#### Vertebrate

Our analysis reveals a clear pattern of gene expansion in CLKs across vertebrate (Fig. 1, blue). Gene duplication is a major mechanism for generating new genetic material and driving evolutionary innovation. Three critical CLK duplication events that produced the canonical CLK1-4 genes present in mammals were identified: (1) the first duplication of the CLK2 ortholog was in earliest diverging vertebrates, lampreys (Hyperoartia), to create the first CLK4 gene (2) then, there was another duplication of the CLK2 ortholog, giving rise to CLK3 orthologous genes in lobe-finned fishes (Sarcopterygii), and finally, (3) duplication of the CLK4 ortholog to create the first CLK1 gene, present only in amniotes (Mammalia and Aves). Despite the strong retention of these genes across vertebrates, we identified two significant loss events.

The first of these CLK duplications, which occurred at the base of vertebrates, coincides with a known whole-genome duplication (WGD) event (58). In the sea lamprey (*Petromyzon marinus*), one of these duplicated genes (CLK2a) clusters within the CLK2 ortholog branch, while the other (CLK2b) aligns with the basal CLK4 position, reinforcing the link between this WGD and the emergence of CLK4. This gene expansion has been largely maintained across vertebrates, with one notable exception: we identified a loss of CLK4 in Neobatrachia, an amphibian lineage that encompasses over 96% of extant frog and toad species (59). Apart from this branch, all other vertebrates retain a CLK4 ortholog.

The second major duplication occurred within lobe-finned fishes (Sarcopterygii), a lineage that underwent significant adaptations associated with the water-to-land transition (60, 61). In this event, the CLK2 gene duplicated again, producing the first CLK3 ortholog, which has been retained in all vertebrate species examined. The widespread conservation of CLK3 across vertebrates suggests it plays an essential functional role. Intriguingly, we also detected a duplication of CLK2 (referred to as CLK2b) in the elephant shark, *Callorhinchus milii*. Despite this species being classified among the cartilaginous fishes (Chondrichthyes), CLK2b clustered phylogenetically close to CLK3 in Sarcopterygii. Interestingly, an independent study places *C*. *milii* as a sister group to Sarcopterygii, lending support to our phylogenetic findings and suggesting that CLK3 may have originated within this lineage. The remaining CLK genes in *C*. *milii* grouped with CLK2/CLK4 orthologs found in other cartilaginous fishes, adding uncertainty about the precise evolutionary origin of CLK2b. This potential phylogenetic misplacement is noteworthy, as *C*. *milii* is widely used as a model organism in vertebrate evolutionary research (62).

The final of these key CLK expansion events was the duplication of CLK4 in amniotes to produce CLK1, representing another key lineage in vertebrate evolution where complete terrestrialisation was achieved. CLK1 and CLK4 are the most closely related in sequence, and even the CDD database groups them together as cd14213 (CLK1_4). Initially, we used this combined profile for our search, then refined our results by generating a phylogenetic tree to separate ortholog families and create unique profiles for CLK1 and CLK4. During this process, we identified some annotation errors where CLK4 genes were mistakenly labeled as CLK1. We only found one publication which has used this misclassification, where an embryonic knockdown of “CLK1” (actually CLK4) in *Xenopus tropicalis* resulted in minor phenotypic changes (7). Using our refined search, we confirmed that CLK1 has been retained across mammals. However, we identified its loss in Psittacopasserae, a large avian lineage comprising over 60% of all bird species, including parrots (Psittaciformes) and songbirds (Passeriformes). At the base of this lineage, we identified a highly divergent CLK1 sequence in *Nestor notabilis*, indicating that a functional shift may have occurred, ultimately rendering the gene nonessential and leading to its loss in descendant species.

Beyond these major duplication events, we also identified three other lineage specific duplications. A duplication of both the CLK2 and CLK4 genes occurred at the base of teleosts (eg zebrafish *Danio rerio*), a division which encompasses 96% of all extant species of ray-finned fishes (Actinopteri). This is likely attributed to the well-known WGD event that occurred in the common ancestor of all teleosts (63, 64). This is supported by the presence of only one copy of CLK2 and CLK4 in *Amia calva* (Amiiformes) and *Lepisosteus oculatus (*Semionotiformes), fish species that diverged just prior to teleosts. Among mammalian species, we identified two independent CLK duplications. A CLK2 duplication was found within all great apes (Hominidae) except *Homo sapiens*. A BLAST search revealed that the human ortholog of this gene is classified as a pseudogene (NCBI Gene ID: 1197), indicating that functional loss occurred following its duplication in the ancestral great ape lineage. Another duplication was of CLK3 in the microbat family, Vespertilionidae. In particular, one of these paralogs has undergone significant divergence, as seen in CLK3b in *Myotis lucifugus*. These findings emphasize that due to functional redundancy, duplications commonly undergo divergence, which may lead to either function gain, pseudogenization, or eventual loss.

The retained CLK paralogs, CLK1-4, within vertebrate have likely shaped transcriptome complexity in these organisms. Previous studies have demonstrated that as organisms evolve toward greater complexity, there is a corresponding rise in both the intricacy of AS and duplications of splicing regulator genes (65, 66, 67). One reason for this is increased regulation of AS, such as that controlled by the CLK family, which offers a source of transcriptional diversity to facilitate adaptation. Although there is general conservation of core spliceosomal proteins, there is a selective expansion of protein families in metazoans that are involved in splicing regulation, including vertebrate-specific duplications of hnRNPs and SRPKs (67). As such, the three major CLK duplication events likely reflect the increase in splicing complexity necessary for the corresponding shifts in eukaryotic evolution.

#### Gene retention—are CLKs essential?

It is widely acknowledged that genes deemed functionally indispensable are less likely to be lost during evolution and therefore retained within the genome (68). Despite the prevalence of CLKs throughout eukaryotes, we have shown that some single-cell organisms have lost CLK genes entirely. On the contrary, we found no evidence of complete CLK gene loss within multicellular eukaryotes. In addition, genetic KO of the well-studied CLK, "DOA" (*Drosophila melanogaster*, fruit fly) results in severely abnormal neural development and embryonic lethality (4, 69). This underscores the potential indispensability of these kinases in more complex organisms.

It is clear that complex organisms are unlikely to survive without any CLK genes, but it is unknown whether loss of individual paralogs would be detrimental to an organism’s survival. Among the four canonical CLK paralogs in vertebrate lineages, we found gene loss of CLK1 in Psittacopasserae, and CLK4 in Neobatrachia, suggesting a lack of biological necessity within these branches. In contrast, we found no instances of CLK2 or CLK3 gene loss across vertebrate lineages, implying that they may be functionally indispensable. Presently, complete transgenic KO mice have only been generated for CLK1 and CLK2, both of which are shown to be viable and fertile in a controlled laboratory environment (70, 71, 72, 73), indicating that despite the preservation of CLK2 across metazoans, mice can survive and reproduce without this gene. One possible explanation is that while essential genes typically support growth and reproduction, “gene essentiality” is context dependent. Some genes are directly essential, impacting an organism's fertility or viability upon removal, while others are indirectly essential, affecting long-term survival (74, 75, 76). As such, CLK2 KO may not immediately compromise survival in mice, but may decrease long-term survival fitness in natural environments. Alternatively, multiple CLK paralogs within an organism could have redundant functions and compensate in the absence of one another.

Although CLK3 has not been knocked out in a mouse model, a compelling study has explored the effect of embryonic knockdown of CLKs in the frog *X*. *tropicalis*. This species possesses CLK2, CLK3, and CLK4 (incorrectly labeled as CLK1) which are coexpressed in neural tissue during early embryogenesis (7). Individual knockdown using translation-blocking morpholino oligonucleotides demonstrate CLK3 is the only ortholog essential for development, leading to a significant reduction in head and eye size. Embryos with a greater CLK3 knockdown efficiency experienced lethality, while knockdown of CLK2 and CLK4 produced only mild phenotypic changes in embryonic development (7). This study is the first to establish an individual CLK paralog as essential for vertebrate life. Furthermore, this severe neural development phenotype shares common features with the embryonically lethal knockdown of DOA in *D*. *melanogaster*, which could implicate a conserved role (4, 69). Despite DOA being an ortholog of CLK2, a plausible explanation could be that when CLK3 originated from the duplication of CLK2, it acquired this developmentally essential function through the process of gene subfunctionalization.

### A conserved network of interacting proteins

To investigate conserved functions of CLK proteins across diverse eukaryotic species, we analyzed known protein–protein interaction (PPI) data to identify interologs—interacting protein pairs in one species whose homologs also interact in another species. We collected CLK PPI data from online databases for human CLK1–4, as well as CLKs in three model organisms: “DOA” in Drosophila *melanogaster* (fruit fly), “LKH1” in *S*. *pombe* (fission yeast), and “KNS1” in *Saccharomyces cerevisiae* (baker’s yeast). Since human CLK1–4 are homologous and share many interaction partners, we merged their PPI into a single dataset comprising 523 interactions. For the model organisms, we retrieved 86 CLK PPIs in *D*. *melanogaster*, 97 in *S*. *pombe*, and 226 in *S*. *cerevisiae (*Table S2).

The differences in numbers of PPI between the species likely reflects both biological and experimental factors, including disparities in the number of coimmunoprecipitation studies conducted on each species and the general trend that more complex organisms have larger interactomes. Research indicates that the human interactome is roughly 10 times larger than that of *D*. *melanogaster* and 25 times larger than that of *S*. *cerevisiae* (77). Despite this, *S*. *cerevisiae* KNS1 exhibits a relatively high number of interactions, which could suggest an expanded functional role for this kinase within baker’s yeast.

To determine whether interactions were conserved, we used the DIOPT (78) tool to score the homology between CLK PPIs across species. This approach allowed us to identify a total of 54 conserved interolog pairs in the four species. Of these, there were 92 unique CLK interacting proteins, 38 of these in human, 19 in *D*. *melanogaster*, 14 in *S*. *pombe*, and 21 in *S*. *cerevisiae (*Table S2).

**Figure 2:**
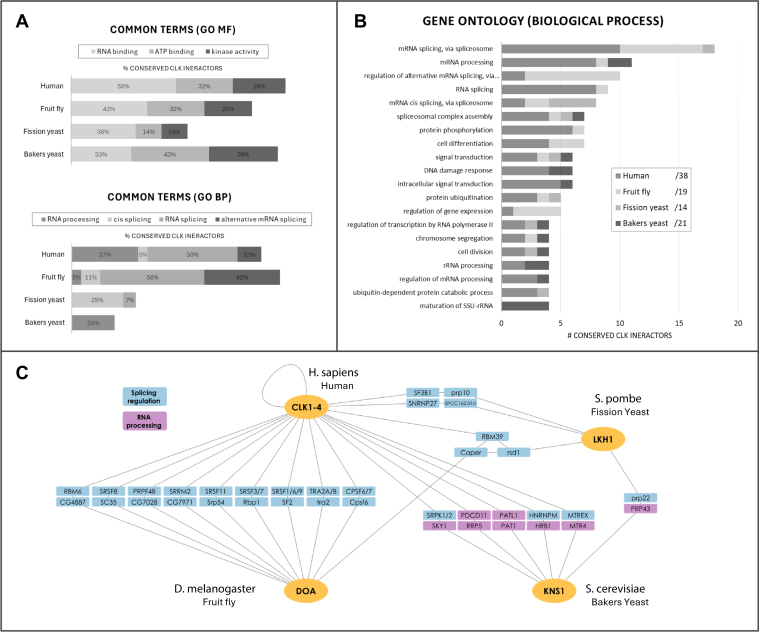
**CLKs have a conserved network of interacting proteins that regulate RNA**. Known CLK protein–protein interactions (PPIs) were collected from online databases for four model species, and homologous interacting pairs (interologs) were identified to compile a list of conserved CLK interactors. Functional information for each conserved CLK interactor was extracted from Gene Ontology (GO). *A*, common terms for GO biological processes (GO BP) and GO molecular function (GO MF) were grouped for each species. *B*, the top 20 GO BP annotations for the interactors are displayed. *C*, a PPI network of conserved CLK interactors that are involved in RNA processing and splicing regulation, as determined by their GO BP annotations. CLK, Cdc2-like kinase.

We then analyzed the list of 92 homologous interactors using the Gene Ontology (GO) knowledgebase (79, 80) to identify shared functions among CLK binding partners. To summarize our findings, we compiled the GO annotations and counted the number of interologs associated with each. These annotations were then categorized based on the most common terms, and we calculated the percentage of interactors corresponding to each category (Fig. 2*A*). For GO molecular functions (MFs), the three most prevalent terms were “RNA binding,” “ATP binding,” and “kinase activity”, with at least one-third of homologous CLK interactors across all species classified as RNA binding (Fig. 2*A*, top). Meanwhile, GO biological process (BP) annotations were strongly skewed toward RNA processing functions (Fig. 2, *A* and *B*). Accordingly, we filtered for GO BP annotations containing the four common terms: “RNA processing,” “cis splicing,” “RNA splicing,” and “alternative mRNA splicing,” (Fig. 2*A*, bottom). In addition, among the top 20 GO BP annotations, RNA-related processes dominated, comprising the top six and accounting for half of the total. The remaining annotations were linked to various cellular functions, including signal transduction, DNA damage response, protein degradation, and chromosome segregation (Fig. 2*B*).

Overall, the results demonstrate that the core, evolutionarily conserved interactors of CLKs are predominantly RNA-binding proteins that regulate either RNA splicing or other RNA processing. To visually demonstrate this, we took the homologous interactors that fall into these categories and generated a PPI network (Fig. 2*C*). Due to gene duplication differences between species, each node is grouped into homologous proteins. In should be noted that, although our analysis does not differentiate whether these RNA-binding proteins are direct phosphorylation targets of CLKs, many have been experimentally confirmed as such (2, 81, 82, 83). Research shows that CLK regulation of AS through phosphorylation of RNA binding proteins is conserved in animals (2, 84) and plants (10, 85) demonstrating that it has maintained this function across vast evolutionary distances. Its presence in the LECA further supports this function being ancestral to this kinase family. Of interest, we observed a shift in the specific RNA processing functions associated with CLKs among different organisms. In human and fruit fly, CLK interactors are linked to the regulation of alternative mRNA splicing, a feature of complex gene regulation (Fig. 2*A*, bottom). In contrast, in fission yeast, the interactors are predominantly associated with cis splicing regulation, and in baker’s yeast, its homologous CLK interactors are primarily connected with general RNA processing rather than splicing (Fig. 2, *A* and *C*).

This functional shift likely reflects the evolutionary differences in splicing complexity: while the LECA is believed to have had a highly complex spliceosome, many eukaryotic lineages—yeasts in particular—have simplified their splicing machinery (65, 86, 87). For instance, only 4% of protein-coding genes in *S*. *cerevisiae* undergo constitutive “cis” splicing, and a mere 0.2% exhibit alternative splicing, predominantly *via* intron retention (88, 89). Moreover, KO of KNS1 in *S*. *cerevisiae* does not alter the pre-mRNA/mRNA ratio (90), further supporting the idea that this CLK does not regulate splicing in baker’s yeast. By contrast, *S*. *pombe* has retained more of its splicing complexity, with 43% of genes containing introns and 4.5% undergoing AS (91). Studies have shown that KO of LKH1 in *S*. *pombe* increases the pre-mRNA/mRNA ratio by eight-fold (92), although it does not affect global AS (82), supporting our findings that its homologous interactors are associated with cis splicing. These findings suggest that in simpler unicellular eukaryotes, CLKs may primarily regulate more basic forms of RNA processing. Nevertheless, the occurrences of CLK regulated AS in plants (10, 85) and indicates that this function is ancestral and has been lost in some yeasts.

Beyond RNA binding proteins, our GO molecular functions analysis (Fig. 2*A*, top) indicates CLKs also interact with other kinases and enzymatically active proteins to influence a variety of cellular processes. CLKs have been shown to both regulate and be regulated by other kinases. For example, human CLK1 is phosphorylated by Akt2 in response to insulin (93). In addition, CLKs phosphorylate other kinases, such as Aurora B, which is targeted by human CLK1, 2, and 4 to regulate the abscission checkpoint (94), and Cmk2 in *S*. *cerevisiae*, which is phosphorylated by KNS1 (90). Our GO BP analysis (Fig. 2*B*) of CLK interactors further supports CLK involvement in diverse pathways, such as the DNA damage response that has been documented for CLKs in human (94) and various species of yeast (54, 55, 57, 90). In addition, roles in chromosome segregation have been reported for human CLK1,2,4 (94), *Drosophila* DOA (95), *S*. *pombe* LKH1 (96, 97), and even in kinetoplastids like *Trypanosoma* and *Leishmania*, where their CLK homologs have integrated into unique kinetochore complexes (38, 39, 40, 41).

### Structural evolution of CLKs

#### Conservation of the kinase domain

**Figure 3:**
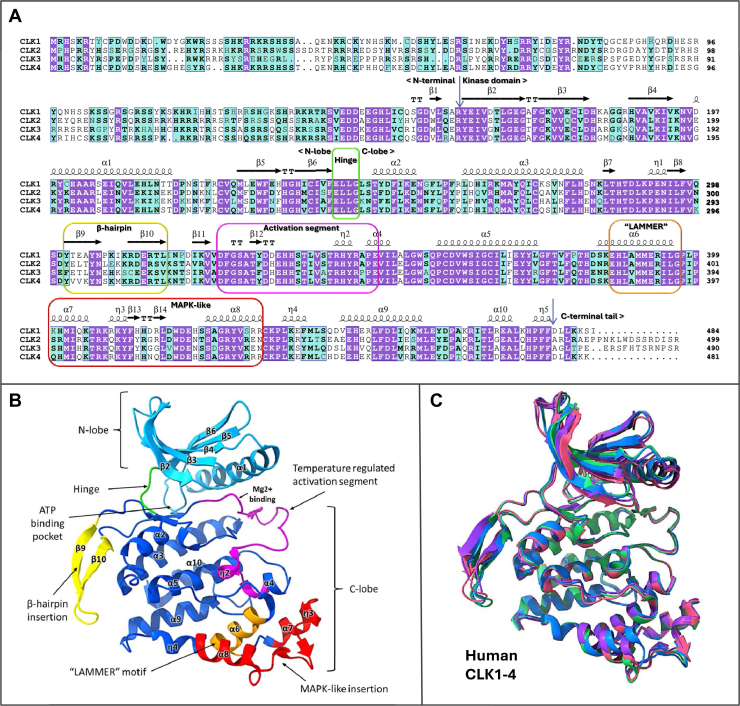
**Sequence alignment and structure of human CLKs compared to diverse eukaryotes**. *A*, amino acid sequences for human CLK1-4 were aligned using MAFFT, with conserved structural elements depicted above the alignment. *Purple boxes* denote strict identity, *while blue boxes* indicate 75% group similarity, with *bold characters* representing amino acids with similar physicochemical properties. α-helices and 3_10_-helices (η) are displayed as big and small squiggles, respectively. β-strands are shown as *arrows*, and strict β-turns as “TT” letters. *B*, crystal structure of CLK2 (PDB:6FYL) kinase domain with structural elements colored and labeled corresponding to those in the sequence alignment. *C*, crystal structure overlay of CLK1-4 kinase domains (CLK1 PDB:6R8J-*pink*, CLK2 PDB:6FYL-*green*, CLK3 PDB:6Z53*blue*, CLK4 PDB:6fyv-*purple*). CLK, Cdc2-like kinase; PDB, Protein Data Bank.

**Figure 4:**
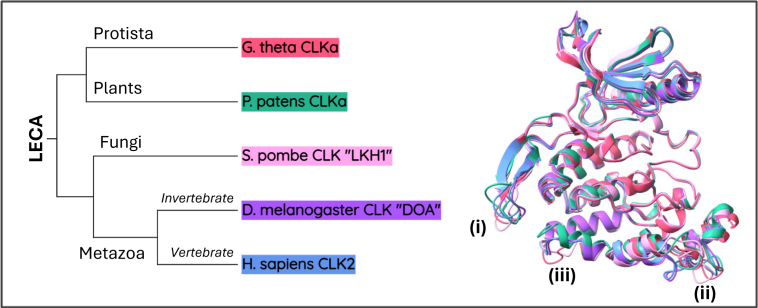
**Structural conservation of CLK proteins traces back to the last eukaryotic common ancestor (LECA)**. *Left*: phylogenetic tree illustrating the relationships among the four major eukaryotic kingdoms and their connection to the LECA. Representative species are color-coded to correspond with their structural models. *Right*: AlphaFold3-predicted structural overlays of CLK proteins from five species spanning the eukaryotic tree of life, highlighting a conserved kinase domain structure likely present in the LECA. Numbered regions (i–iii) indicate areas of structural divergence. CLK, Cdc2-like kinase.

**Figure 5:**
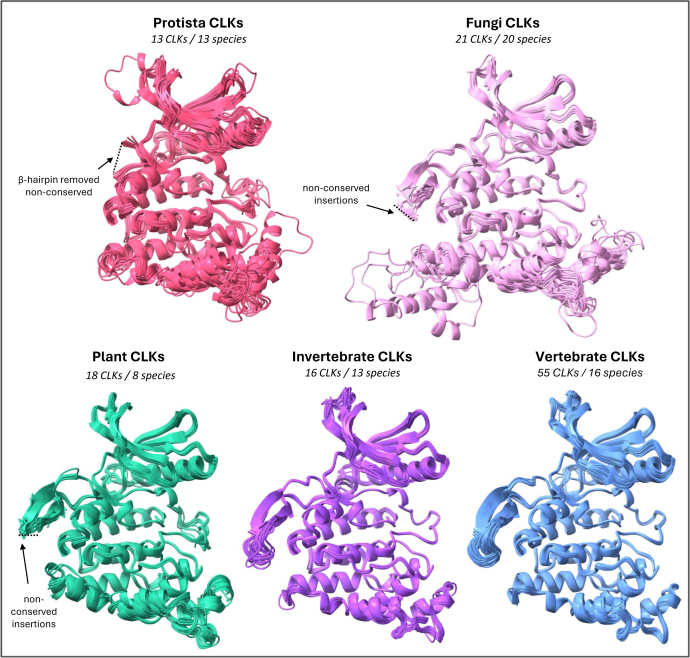
**Structural overlays of diverse CLK kinase domains across eukaryotes reveal both conserved and divergent features**. AlphaFold3-predicted CLK structures from all four major eukaryotic kingdoms are shown: protista (*red*), fungi (*pink*), plants (*green*), and metazoa, further divided into invertebrates (*purple*) and vertebrates (*blue*). To improve visualization, large nonconserved insertions in β-hairpins in the protista, plant, and fungal structures were trimmed, as indicated by *dashed lines*. See Supporting Figure S1 for complete structures alongside pLDDT values. CLK, Cdc2-like kinase.

**Figure 6:**
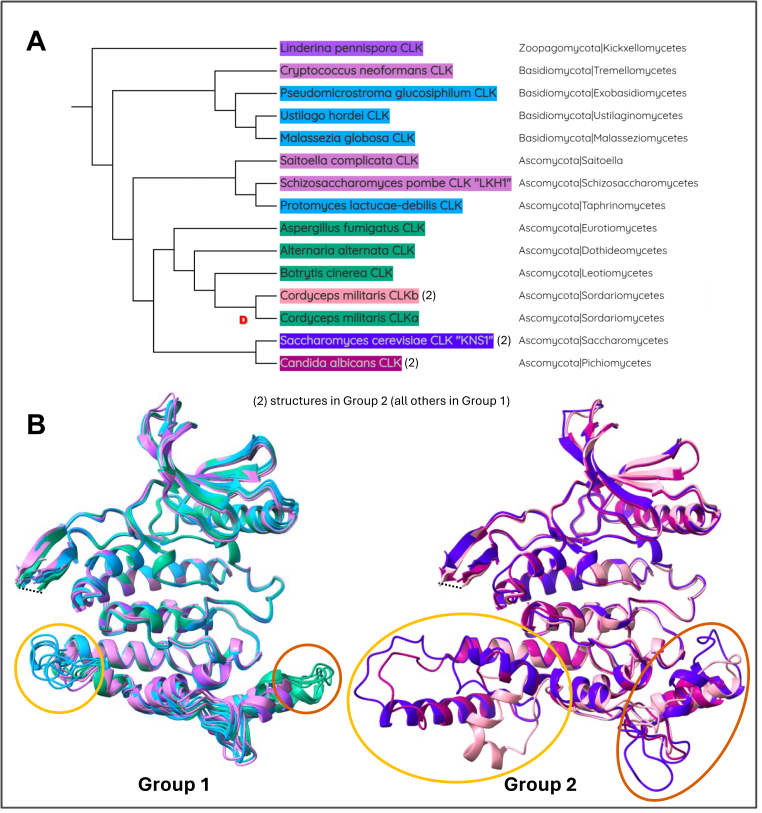
**Structural diversification of CLKs in the Fungi kingdom**. *A*, phylogenetic tree of fungal species with color coordination to the structures below. The *red* “D” indicates a duplication event. *B*, overlays of AlphaFold3 predicted structures of CLK kinase domains from various fungal species. Groups 1 and 2 have been separated to best display the structural deviations. The *yellow circles* and *orange circles* demonstrate two common regions where diversification has occurred. The *dashed lines* indicate nonconserved insertions in the β-hairpin that were removed. CLK, Cdc2-like kinase.

To gain more insight into the functional evolution of CLKs across eukaryotic evolution, we have performed structural comparisons of their kinase domains. Advancements in artificial intelligence-based protein structural predictions using AlphaFold3 (98) have allowed us to generate high-confidence models of CLK kinase domains across all eukaryotic kingdoms (Figure 4, Figure 5, Figure 6). All CLK homologs consist of two primary regions: a conserved kinase domain and an intrinsically disordered N-terminal region. Due to the disordered nature of the N terminus, our structural analysis focuses on the kinase domain. Although crystal structures of human CLK1–4 have previously been compared (1, 2, 16, 17), our work builds on this by annotating the sequence alignment with their structural features (Fig. 3, *A*–*C*).

Human CLK1-4 demonstrate a high degree of conservation of the kinase domain and divergence of their N termini, as shown by a sequence and structural alignment of human CLK1-4 (Fig. 3, *A* and *C*). These enzymes display typical kinase features, including an ATP binding pocket (Fig. 3*B*) situated within a hinge region (Fig. 3, *A* and *B*, green) linking the N- and C-lobes of the protein. The N-lobe comprises six β-strands and one α-helix, while the C-lobe is made up of 15 helices (α or 3_10_), a β-hairpin, and six short β-strands (Fig. 3, *A* and *B*). Within the α6 helix, which is located within the C-lobe of CLK1-4, is the well-known “EHLAMMERILG” motif (Fig. 3, *A* and *B*, orange). Preceding this motif is a distinct MAPK-like insertion which keeps the α6 helix inaccessible to solvents (Fig. 3, *A* and *B*, red) (99). In addition, this group of enzymes possess a unique insertion at the beginning of the C-lobe, forming an extended β-hairpin structure (Fig. 3, *A* and *B*, yellow). The C-lobe of CLKs contain the activation segment (Fig. 3, *A* and *B*, represented in magenta) positioned in front of the ATP binding pocket, which, as discussed below, is subject to unique temperature regulation.

These annotated structural features provide a foundation for interpreting CLK homologs in other taxa. The kinase domain’s conservation among human CLK1–4, which diverged following a duplication event approximately 500 million years ago at the base of vertebrates (100), underscores its critical role (Fig. 3*C*). Extending our comparisons to other eukaryotes, we find that this structural conservation reaches back ∼1600 million years ago to the LECA, with conserved homologs present in all major eukaryotic kingdoms (Fig. 4). This deep conservation suggests that the CLK present in the LECA likely performed similar biological functions as modern CLKs. Evidently, CLK-mediated phosphorylation of SR proteins to regulate AS is highly conserved in both animals (2, 84) and plants (10, 85).

Despite overall conservation, three consistent regions of divergence were noted: (1) between β9 and β10 in the β-hairpin, (2) the MAPK-like insertion between α7 and α8, and (3) the region between α9 and η4 (Figs. 3*B* and 4). Interestingly, even in structurally divergent CLKs, these regions remain the primary sites of variation, implying that they are hotspots for functional adaptation. This aligns with evolutionary trends in unicellular eukaryotes toward genome reduction, where complex processes like AS are often simplified (65, 86, 87, 101, 102).

#### Diversification of the kinase domain

Although CLK structures are highly conserved among invertebrate and vertebrate species, notable divergence occurs in the other three eukaryotic kingdoms (Fig. 5). These structural differences are concentrated in the regions noted in Figure 4 (i)-(iii), and also the activation segment (discussed below). The most striking divergence involves large insertions in the β-hairpin region. In plant and fungal CLKs, although the two core β-strands of the β-hairpin resemble those of metazoans, variable regions are present between them (pink/green, dashed lines, Fig. 5). In protista, the β-hairpin is inconsistently retained—replaced in some species by large regions that were omitted to improve visualization (red, Fig. 5). The complete structural predictions along with their pLDDT values can be seen in Figure S1*A*.

To understand the function of the β-hairpin and its variability, we compared CLKs to the closely related serine/arginine protein kinase (SRPK) family. Like CLKs, SRPKs phosphorylate SR proteins, but utilizing a unique intrinsically disordered insertion at the corresponding β-hairpin position known as the spacer insert domain (SID) (103, 104). This 270-residue segment in human SRPK1 is noncatalytic, yet functionally important. By interacting with SRPK1’s N terminus, the SID accelerates SR protein phosphorylation by enhancing ADP release. Although SRPK1 can still phosphorylate serine/arginine-rich splicing factor1 (SRSF1) without the SID, its efficiency drops over tenfold (103, 104). Thus, this region significantly impacts functionality, suggesting that β-hairpin insertions in CLKs may serve similar regulatory roles. Its divergence likely reflects species-specific adaptations in protista, plants, and fungi.

To explore structural divergence in fungi, we overlaid kinase domain structures from 15 fungal CLKs representing 14 species, grouped by structural similarity rather than phylogeny (Fig. 6). In basal Zoopagomycota (purple, Fig. 6, *A* and *B*) and both Basidiomycota and Ascomycota (pink), CLKs closely resemble the ancestral LECA structure. The two main regions of divergence in fungi map to the conserved hotspots (ii) and (iii) and are marked in orange and yellow, respectively (Fig. 6*B*). The orange-circled MAPK-like region shows variation across Ascomycota species (Group 1, green, and Group 2), which are also observed in protista (red, Fig. 5). Interestingly, this region contains a known splice site conserved from *Drosophila* (105) to humans, suggesting that insertions may have evolved from AS or intron retention.

The yellow-circled region contains insertions that appear to have evolved independently on at least four occasions within Basidiomycota and Ascomycota lineages (Group 1, blue, and all of Group 2). One of the most structurally divergent CLKs is KNS1 from *S*. *cerevisiae*, which contains a large insertion at this site (indigo, Fig. 6). Unlike its counterpart LKH1 in *S*. *pombe*, KNS1 does not interact with splicing regulators, reflecting the broader simplification of the splicing machinery in this yeast. The unique structural features of KNS1 likely represent a functional shift, consistent with its loss of splicing-related roles. Nevertheless, *in vitro* studies show that KNS1 retains the ability to interact with and phosphorylate SR proteins from other species (81), emphasizing that divergence in structure reflects adaptation rather than loss of catalytic capability toward these main substrates.

#### The disordered N terminal

**Figure 7:**
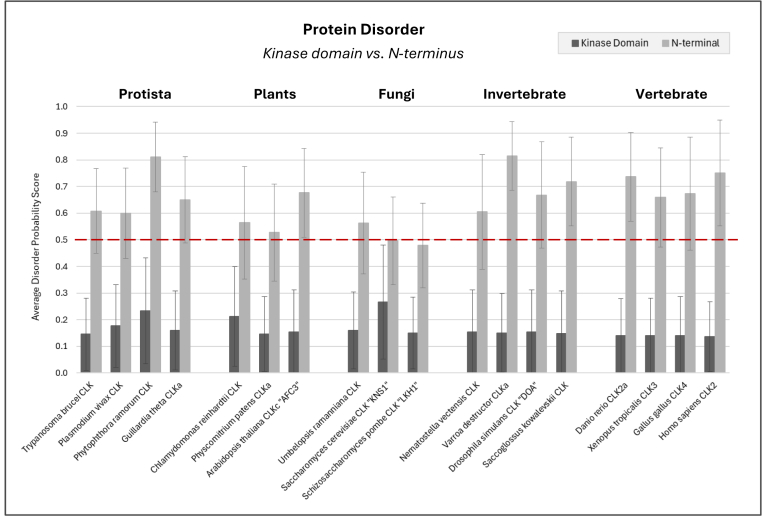
**Intrinsic disorder is a conserved feature of the N termini in CLK proteins across eukaryotes**. CLK sequences from a range of eukaryotic species were segmented into the N-terminal region and the kinase domain. Disorder probability for each residue was calculated using PrDOS (Protein Disorder Prediction Server). Residues with scores above 0.5 (indicated by the *red line*) are predicted to be disordered. Mean disorder scores were calculated for each region, with error bars indicating standard deviation. CLK, Cdc2-like kinase.

Although the kinase domain of CLKs can be structurally characterized, the N terminus prevents such analysis due to its intrinsically disordered and unstructured nature. To examine this further, we utilized the protein disorder prediction server (PrDOS) (106). This tool assigns a probability score to each amino acid residue, with scores above 0.5 indicating disorder. We calculated the overall disorder for both the N terminal and kinase domain by averaging the scores across each region (Fig. 7). Full-length protein disorder profiles are provided in Figure S2. The data confirm that N-terminal disorder is a conserved feature of CLKs across eukaryotes. Notably, metazoan CLKs tend to exhibit higher levels of disorder compared to those from fungal species. Despite their low sequence conservation, expression of the N terminus alone can bind SR proteins from diverse organisms, reinforcing a conserved functional role (81). These results support the broader observation that homologous intrinsically disordered regions often diverge in sequence while retaining functional properties across species (107).

Although the intrinsically disordered nature of CLK N termini is conserved, sequence variation within this region allows for species-specific regulation of substrate specificity, kinase activity, and subcellular localization. A key adaptation is the increased abundance of arginine-serine (RS) motifs, which are more prevalent in metazoans (Fig. S3). When present in high numbers, these motifs form RS domains, which preferentially associate with other RS domains—a characteristic shared with their SR protein substrates. In mammals, the abundance of RS motifs promotes CLK self-association, resulting in oligomerization and increased binding affinity for SR protein targets (108, 109).

CLKs are also differentially regulated through autophosphorylation of their N termini to mediate kinase activity. This is maintained in diverse organisms, including yeast, mammals, plants, and fruit fly, however, the specific phosphorylated sites are highly variable between CLKs (14, 19, 110, 111, 112, 113). Furthermore, individual N termini from CLKs undergo differential phosphorylation to control their activity and specificity. Research by Prasad and Manley (83) found that the pattern of autophosphorylation on CLK1 regulates kinase activity and specificity toward its SR protein substrates. Herein, they demonstrated that (1) autophosphorylation of CLK1 on tyrosine residues (but not serine/threonine) dictates specificity toward SRSF1 (2), autophosphorylation of CLK1 on serine/threonine residues dictates specificity toward SRSF2, and (3) phosphorylation of SRSF5 remains unaffected by the pattern of CLK1 autophosphorylation (83).

The importance of the N-terminal domain regarding subcellular localization has been highlighted in two model organisms, namely *D*. *melanogaster* and *Caenorhabditis elegans*. The CLKs within these organisms are alternatively spliced to produce either nuclear-specific, or cytoplasmic-specific isoforms, with the only distinguishing factor being a difference in their N termini (5, 105, 114). In both species, these distinct isoforms perform unique functions within their specific subcellular compartments.

### Unique thermoregulation of CLKs

#### The temperature-regulated activation segment

**Figure 8:**
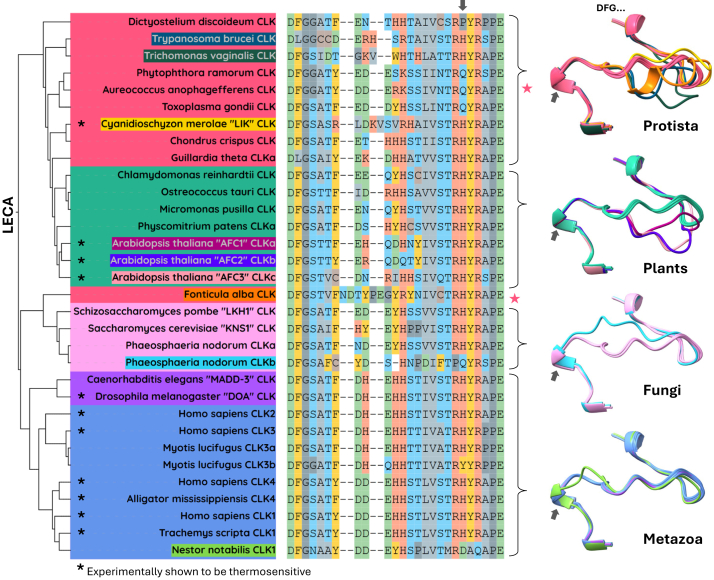
**Conservation and diversification of the temperature-regulated activation segment in CLK proteins across eukaryotes**. A phylogenetic tree illustrating the relationships among CLK proteins from the four major eukaryotic kingdoms. Adjacent to each CLK on the tree are aligned activation segment sequences. AlphaFold-3 predicted structural overlays are shown on the right and are color-coded to match their corresponding CLKs in the tree. *Asterisks* indicate kinases that have been experimentally confirmed to exhibit thermosensitivity. *Gray arrows* highlight the critical histidine residue associated with CLK thermal responsiveness. Amino acids are colored based on their physicochemical properties. CLK, Cdc2-like kinase

Like in other kinases, the activation segment of CLK is located in front of the ATP binding pocket (Fig. 3*B*, magenta). The ATP-coordinating magnesium ion directly interacts with the DFG loop, facilitating the transfer of the γ-phosphate to specific substrates. This domain undergoes a conformational change to regulate accessibility and positioning of the nucleotide which, for many kinases, is brought about through phosphorylation of this region (115). However, in the case of CLKs, the activation segment is also thermally regulated, typically in a reversible manner (6). This thermal regulation is particularly unique for CLKs, which display a negative enzymatic Q_10_ temperature coefficient. The Q_10_ for most mesophilic enzymes equals 2 to 3, meaning that for each 10 °C rise above their physiological temperature, their reaction rate doubles or triples (116). Instead, human CLK1 and CLK4 are minimally active at 38 °C and increase their activity ∼4-fold upon cooling to 35 °C (6). Pivotal to this function is the P + 1 loop, an important site for substrate interaction within the activation segment of most kinases (Fig. 3*B*, η2). A single histidine (H) within this loop underlies CLK thermoregulation: its replacement by glutamine (Q), as in SRPK1, eliminates CLK1’s temperature sensitivity, whereas introducing histidine into SRPK1 confers temperature dependence (6). This underscores the critical role of a single histidine in CLK thermoregulation (gray arrows, Fig. 8).

To understand how this unique regulatory mechanism evolved, we examined the conservation of the activation segment across eukaryotes using phylogenetic, sequence, and structural analyses (Fig. 8, Fig. S3). Although there is structural conservation of the activation segment across all four eukaryotic kingdoms, certain CLKs display notable divergence. However, these altered regions typically have lower pLDDT values, and therefore their true structures may differ from those modeled (Fig. S1*B*). Sequence comparisons demonstrate that the first six and last eight amino acids of the activation segment are highly conserved, while the central region shows greater variability (Fig. S3). However, the critical histidine remains largely conserved, with only five unique mutation events detected. Such conservation, alongside experimental evidence of temperature sensitivity in mammals (6), *D*. *melanogaster* (6), *A*. *thaliana* (10, 24), *Trachemys scripta* (6), *Alligator mississippiensis* (6), and *C*. *merolae* (23), supports an ancient origin for this feature, likely present in the LECA (asterisks, Fig. 8).

##### Protista

Despite extensive amino acid substitutions, many protist species maintain the conserved structural framework of the activation segment (protista, red, Fig. 8). In protista, two notable mutations of the critical histidine occur, in TSAR protists (H→Q) and Amoebozoa (H→P), suggesting a loss of temperature sensitivity. Furthermore, while TSAR group members and *Dictyostelium discoideum* retain conserved structural features, *E*. *histolytica* exhibits both significant sequence (Fig. S3) and structural (not shown) deviations. This supports the notation that loss of CLK function in *Entamoeba* preceded the loss of this gene observed in *A*. *castellanii* (Fig. 1).

Certain protists, like *Trypanosoma brucei* (protista, dark green, Fig. 8) and *Trichomonas vaginalis (*protista, dark blue, Fig. 8), exhibit structural divergence due to substitutions in the central activation segment, but importantly, not the critical histidine needed for temperature sensitivity. In *T*. *brucei*, these changes may reflect its adapted function within Kinetoplasts as a component of the kinetochore (38, 39, 40, 41).

*C*. *merolae* (protista, yellow, Fig. 8) and *F*. *alba* (protista, orange, Fig. 8) have an additional two and three amino acids, respectively, in their activation segments, resulting in altered structures. Among protists, *C*. *merolae* is the only species in which CLK thermosensitivity has been experimentally tested. This organism thrives in hot springs (up to 56 °C) and its thermophilic CLK, named “LIK”, has peak activity at ∼48 °C and inhibition at 56 °C. As such, it is possible that the altered activation segment in this species may contribute to thermal stability of the kinase. Despite this intriguing observation, the amino acid insertions in *F*. *alba* do not contribute to extreme living conditions as this organism only grows within a temperature range of ∼16-37 ^o^C (117).

##### Plants

Activation segment conservation is strong among early plant lineages, including green algae and *P*. *patens* (plants, green, Fig. 8). However, angiosperms display significant structural diversification in their activation segments. The three CLKs present in *A*. *thaliana* all have unique structures, each differing by the orientation of the central region of the activation segment (plants, AFC1-3 Fig. 8). Previous work identified AFC2 as thermosensitive (10). In our recent study (unpublished, available as a preprint (24)), we found that all three AFCs are thermosensitive and rapidly lose activity above 32 °C. Despite sharing this upper thermal limit, they exhibit distinct temperature activity profiles: AFC1 is most active at 4 to 20 °C, AFC2 at 4 to 28 °C, and AFC3 at 24 to 32 °C. Although variability within their activation segments may contribute, mutational analyses suggest that additional regions, such as the N terminus, also play a role in regulating temperature sensitivity (24).

##### Fungi

Fungal CLKs are largely conserved in both structure and the thermosensitive histidine (fungi, pink, Fig. 8). The only exception is *Phaeosphaeria nodorum*, where a gene duplication event yielded two isoforms: CLKa and CLKb. While CLKa retains the ancestral structure, CLKb diverged significantly, including loss of the critical histidine. This example highlights a common evolutionary trend in duplicated genes, where functional redundancy often permits divergence, potentially resulting in neofunctionalization, pseudogenization, or eventual gene loss.

##### Metazoa

Metazoan CLKs are the most conserved overall in both sequence and structure, with two exceptions (metazoa, purple/blue, Fig. 8). First, in the bat *M*. *lucifugus*, a duplication of CLK3 produced two isoforms: CLK3a, which retains an activation segment identical to human CLK3, and CLK3b, which carries three amino acid changes—including the critical histidine (H→Y). This mirrors the situation in *P*. *nordum*’s CLKb, illustrating how gene duplication can drive functional divergence or pseudogenization. The second exception occurs in the early diverging passerine bird *N*. *notabilis*, where CLK1 displays structural variation and lacks the critical histidine. This alteration may represent an intermediate step preceding the complete loss of this gene observed in other passerine lineages (Fig. 1).

#### Adaptable temperature regulation

The temperature activity profiles of CLKs are remarkably adaptable, both for orthologs between species, and for paralogs in the same organism. This is an important feature that enables CLKs to adapt their thermal properties to match an organism’s physiological temperature range, as seen for “LIK” in the thermophile *C*. *merolae* (6, 10, 23). Multiple CLKs present in the same species can also adopt distinct thermal profiles, such as AFC1-3 in *A*. *thaliana* (24).

In humans, CLK1 and CLK4 show maximal activity at 22 to 24 °C but become inhibited at 38 °C, corresponding to the upper limit of human physiological temperature (6). We have also experimentally found that CLK2 and CLK3 follow similar patterns, although with around 1 °C and 2 °C lower inactivation temperatures, respectively (unpublished). In reptiles, CLK1 (*T*. *scripta*) and CLK4 (*Alligator mississippiensis*) also display temperature-dependent regulation, with peak activity at 25 °C and inhibition at 35 °C, despite possessing activation segments identical to their human orthologs (6). Similarly, the *D*. *melanogaster* CLK “DOA” has an activation segment identical to human CLK2, yet is inhibited at 32 °C and most active at 20 °C.

These observations indicate that CLK thermal profiles are determined by regions outside the activation segment, which are highly adaptable across and within species. A likely contributor is the intrinsically disordered N terminus, as its deletion in human CLK1 and CLK4 results in modest alterations to their temperature–activity profiles (6).

#### Temperature controlled biological functions

The unique thermosensitivity of CLKs adds an intriguing dimension to their role in the regulation of AS. Aside from generating novel protein isoforms, AS can modulate gene expression by producing “poison” transcripts that undergo mRNA decay (118). It is through this mechanism that body temperature controlled AS profoundly shapes global gene expression, having evolutionary roots deeper than the core circadian clock itself. Of significance, the family of SR proteins across diverse eukaryotes is subject to regulation in this manner, modulating their expression in a temperature-dependent fashion and establishing a feedback loop for AS-linked mRNA decay (118). CLKs influence a large proportion of the temperature-dependant transcriptome, regulating over 50% of thermosensitive exons *in vitro* (6, 10, 25). Physiological functions linked to the temperature control of CLKs include mammalian circadian rhythms (6, 25), reptilian temperature-dependent sex determination (TSD) (6) and plant thermomorphogenesis (10).

Circadian rhythms operate on a ∼24 h cycle, coordinating the cyclic expression of genes to regulate organism physiology over the course of a solar day. These systems are observed across diverse organisms, spanning from mammals to plants, with light and temperature serving as two major universal timing cues (119). In response to light, mammals adjust their body temperature by 1 to 4 °C in day-night cycles, which is accompanied by rhythmic AS through altered phosphorylation of SR proteins (25, 120, 121, 122). For instance, the expression of cold-inducible RNA-binding protein (CIRBP) oscillates in a temperature-dependent manner, regulating many important circadian mRNAs, including “CLOCK” (122)). This rhythmic expression of CIRBP is controlled by CLK-dependent AS *via* differential exon inclusion generating a poison transcript (6, 123). In mice, decreased core body temperature (day) results in exon 7a inclusion, producing the full-length CIRBP transcript, whereas increased core body temperature (night) leads to exon 7b/8 inclusion, triggering nonsense-mediated decay.

Due to their conserved thermosensitivity and role in regulating AS, CLKs may contribute to temperature-dependent circadian rhythm control in other eukaryotic organisms, not just mammals. Temperature-dependant AS of circadian genes has been observed in fish (124), yeast (125, 126), plants (127), and fruit fly (128, 129). In *Drosophila*, for example, the circadian clock is influenced by temperature-dependent AS of “TIM” (129), a protein sharing homology with an isoform of mammalian U2af26 that includes exons 6/7 (U2af26Δ67) (130). Both interact with and affect the stability of "PERIOD" homologs in their respective species, demonstrating shared functionality. Notably, U2af26Δ67 undergoes rhythmic splicing in a CLK-dependent manner due to circadian temperature fluctuations (121). As such, it is tempting to speculate that the CLK homolog “DOA” might participate in temperature-mediated AS of TIM to regulate the circadian cycle in *Drosophila*.

Across diverse reptiles, sex is determined by the temperature at which their eggs are incubated. In the turtle *T*. *scripta elegans*, embryonic development at 26 °C produces all males, while those incubated above 31 °C, produces all females (131). At temperatures in between, the broods will give rise to individuals of both sexes. Although the mechanisms of TSD are not well understood, researchers have suggested that AS of the polycomb-repressive complex 2 component "JARID2" may play a role. Herein, males produced at 26 °C preferentially retain intron 15 (132). *In vitro* experiments indicate that inhibiting CLKs at 26 °C reduces intron 15 retention of JARID2, suggesting the involvement of this kinase family in TSD (6). Furthermore, CLK1 in *T*. *scripta* has full activity below 26 °C, and significant (∼90%) reduction above 31 °C, essentially representing an on-off switch for TSD in this species.

In Arabidopsis, CLKs (AFC1-3) have been implicated in both low-temperature acclimation and thermomorphogenesis (10, 24, 133). Although earlier studies reported that KO of AFC2 leads to an exaggerated high-temperature phenotype (10), we were unable to reproduce this result in a recent unpublished study (24). Instead, our findings showed that either KO of all three AFCs or their chemical inhibition with TG003 reduced high temperature–induced hypocotyl elongation, indicating a positive role for CLKs in this process.

In the fission yeast, *S*. *pombe*, deletion of its CLK “LKH1” leads to temperature-dependant changes in poly (A) + mRNA localization (92). In LKH1 KO cells grown at 36 °C, 23% of the cells exhibit nuclear accumulation of mRNA, while at 30 °C, 70% of the cells show cytoplasmic clustering of mRNA. In contrast, the mRNA in wild-type cells grown at these temperatures is uniformly distributed throughout the nucleus and cytoplasm. This observation suggests CLKs regulate mRNA subcellular distribution, which could potentially occur through their SR protein substrates, which have a critical function in mRNA export (134). In a second yeast species, *Candida albicans*, KO of its CLK “KNS1” revealed a role in dimorphic transitioning (55). Interestingly, higher incubation temperatures, which would reduce KNS1 activity, are known to increase the occurrence of the yeast-to-hyphae transitioning, suggesting this kinase may regulate the morphogenesis of *C*. *albicans* in a temperature-dependent manner (135, 136). Finally, the Σ1278b filamentous strain of *S*. *cerevisiae*, exhibits temperature-sensitive defects in filamentous growth. Remarkably, this can be overcome by either knocking out KNS1, or by overexpressing individual genes downstream of the MAPK signaling pathway to activate filamentous growth (137). As such, temperature dependent control of KNS1 activity may be a negative regulator of flocculation *via* the MAPK pathway in this species.

The physiological functions of CLK are quite diverse. However, the question whether functionality is exclusively controlled through changes in AS and SR protein phosphorylation, or whether other phosphorylation targets and mechanisms exist, remains to be solved. What is clear is that CLKs regulate a significant portion of the temperature-dependent transcriptome, and that this function is likely ancestral to eukaryotes. Therefore, while the full implications of the CLK family being thermosensitive are not yet fully understood, this area of research holds significant promise for new insights and innovation.

## Concluding remarks

This study expands on our understanding of the evolution and diversification of the CLK family. Although some unicellular eukaryotes have lost CLKs entirely, all multicellular organisms possess at least one member in this family. This suggests they are essential genes in more complex organisms. CLK homologs exhibit strong functional conservation in regulating alternative splicing, phosphorylation of SR proteins, and responding to temperature changes. However, they have also adapted their functionality and thermosensitivity in a species-specific manner to meet the requirements of diverse organisms living at different temperatures.

The intricate interplay between CLKs and their substrates governs temperature-dependent gene expression programs, highlighting the complexity of eukaryotic regulatory networks. As we comprehend the molecular implications of the newly found temperature regulation of CLKs, we realize how much there is to uncover about their biology. Understanding how CLKs integrate environmental and internal cues to modulate gene expression programs will deepen our understanding of eukaryotic biology. Throughout eukaryogenesis and early eukaryotic evolution, the ability to respond to temperature likely played a key role in adaptation and survival, helping shape the dynamic landscape of gene expression in eukaryotes.

As CLK research sheds light on thermoregulatory mechanisms in diverse organisms, this may offer insight into how they will respond to climate change, which would aid environmental conservation efforts. In addition, our findings have significant implications for understanding CLK biology in human health. The association of CLK dysregulation with various human diseases underscores the importance of further elucidating their functional roles and potential as therapeutic targets and possible application of thermotherapy. Moreover, our study provides valuable insights for studying CLKs in model organisms and their relevance to human biology.

## Experimental Procedures

### Phylogenetic analysis

CLKs were identified using NCBI’s CDD. Four profiles were retrieved, including cd14134 (CLK)–encompassing CLKs from all eukaryotes excluding vertebrates, and cd14213 (CLK1_4), cd14214 (CLK3), and cd14215 (CLK2), which were exclusive to vertebrate species. These CDDs were searched on the UniProt server and individual protein sequences were extracted, along with species and taxonomy information. NCBI RefSeq eukaryotic protein databases were downloaded in the following six groups: protista, plant, fungi, invertebrate, vertebrate_other, and vertebrate_mammal (release #228). The CLK proteins extracted from UniProt were grouped based on their CDD and their taxonomy as per the RefSeq categories. The CDD portion of the sequences was aligned using MAFFT (138) and cropped to the region of the CDD, which is the kinase domain plus 13 additional amino acids at the start. Using these alignments, HMMER v3.1b2 (http://hmmer.org/) was used to generate HMM profiles and then searched in their respective databases. To increase the power of our analysis by considering more diverged CLKs and those missing CDD annotation, the search results (1 domain E-value >1E-100) were realigned and a new set of HMM profiles were generated and re-searched. In addition, to generate unique HMM profiles for CLK1 and CLK4, proteins matching cd14213 (PKc_CLK1_4) were differentiated using a maximum likelihood tree generated with MEGA11 (139). Final HMMER search results were obtained and then filtered through reverse searching (phmmer) against the human RefSeq database. Results that matched proteins other than CLKs were excluded. This list demonstrated that above a 1 domain E-value of 1E-80 was generally not a CLK, was highly diverged, or an incomplete sequence. BLASTP was utilized to confirm CLKs with high E-values in species where no other CLKs were present. Bacteria and Archaea databases were also downloaded from RefSeq (release #224) and searched with CLK HMM profiles. On the top results we used BLASTP searches against “Eukaryota” and “*H*. *sapiens*” databases to check for CLK homology.

As we were interested in gene gain and loss events, the dataset was manually curated to differentiate between errors and true genomic events. With the known prevalence of database errors, we proceeded through this part of the analysis with caution. Within our dataset, if a CLK gene gain was identified, this was further investigated by checking genomic locations and sequence alignments. If the suspected duplicated genes were overlapping or directly adjacent, and the sequences were close to identical (3 amino acids difference or less), these were excluded. In addition, organisms found to have additional CLKs that were known to be polyploid were not included in the phylogenetic analysis. In cases where CLK gene loss was seen, results with higher E-values were checked to identify partial sequences or highly diverged CLKs. If nothing was identified, a BLASTP search using the nonredundant protein sequences (nr) database was performed, which in some cases found the missing CLK. Generally, if multiple species within the same taxonomic rank possessed the same gain or loss event, this was a good indication of a true result. Additionally, both gain and loss events were excluded if another species within the same Genus or Family did not match the result. Complete results and specific inclusions/exclusions can be found in Table S1.

Due to the large dataset obtained, representative species were selected for phylogenetic tree generation. Sequences were cropped to the coordinates that had aligned with the HMM profile from the search. The large evolutionary distance of these made sequence alignment difficult. For this reason, we used a structure based sequence alignment method, 3D-coffee (32) and performed initial separate alignments for the four eukaryotic kingdoms. Structures used for the sequence alignments were obtained from either uniport (Alphafold2) or manually processed (Alphafold3). We had matching structures for all protista (19), fungi (27) and plant (19) sequences, and for metazoa, we had 36 structures for the 114 total sequences due to the conserved nature of this group. The four 3D-coffee alignments were manually trimmed to remove poorly aligned regions, and then combined into one alignment using the MAFFT (138) MSA merge tool. A total of 149 sequences across 86 species were aligned using 3D-coffee. A maximum likelihood gene tree with 200 bootstraps was generated using RAxML-NG (140)). In addition we generated a species tree using Timetree (100). We then used GeneRax phylogenetic software (141) which utilizes a species-tree-aware maximum likelihood algorithm, accounting for genomic events such as duplication and loss. For the construction of the final phylogenetic tree, the sequence alignment, gene tree, and species tree were run through GeneRax using UndatedDL reconciliation and LG + G substitution. The final tree was formatted using iTOL (142).

### Conserved protein–protein interactions

We began by downloading known PPIs involving CLK proteins from four model organisms—Human, *D*. *melanogaster* (fruit fly), *S*. *pombe* (fission yeast), and *Saccharomyces cerevisiae* (baker's yeast)—using BioGRID (143), as well as species-specific databases including SGD, DroID, and PomBase. To identify homologous relationships among CLK interactors across these species, we used the DIPOT (78) ortholog finder. Since our focus was on overall homology rather than strict orthology, we applied weighted scores with a moderately relaxed threshold (∼5) and merged human CLK1–4 interactors to eliminate redundant interactions.

To compile our final list of conserved CLK interactors, we utilized the ID mapping tool on the UniProt server, extracting relevant data including GO (biological process), GO (molecular function) and Function [CC]. We used the GO information to find common annotations between the CLK interactors.

### Structural comparisons and disorder prediction

Crystal structures for human CLK1-4 were obtained from the Protein Data Bank (PDB), with the following accession codes: CLK1 (PDB: 6R8J), CLK2 (PDB: 6FYL), CLK3 (PDB: 6Z53), and CLK4 (PDB: 6FYV). A MAFFT (138) alignment was uploaded to ESPript (144) along with their PDB files to align structural features.

CLK kinase domain structures in Figures 4–6, 8 were generated using AlphaFold3 (98). Subsequently, the structures of the kinase domains or activation segments were superimposed and colored using ChimeraX (145). The list of CLK structures used for Figure 5 can be found in Table S3.

To assess protein disorder, we used the PrDOS (Protein Disorder Prediction Server) (106). As we were particularly interested in comparing disorder between the N-terminal region and the kinase domain, each of these regions were analyzed separately to calculate mean disorder. Protein disorder graphs were then generated for full-length sequences.

## Data availability

This article contains supporting information. All supplementary files discussed in the text can be downloaded, and in addition, the sequence alignment and newick file used to generate Figure 1. Please contact the corresponding author if further data are requested.

## Supporting information

This article contains supporting information.

## Declaration of Generative AI and AI-Assisted Technologies in the Writing Process

We also acknowledge the use of OpenAI’s ChatGPT for assistance with grammar, proofreading, and language refinement during manuscript preparation.

## Conflict of interests

The authors declare that they have no conflicts of interest with the contents of this article.

## References

[1] Martín Moyano P., Němec V., Paruch K. (2020). Cdc-like kinases (CLKs): biology, chemical probes, and therapeutic potential. Int. J. Mol. Sci..

[2] Song M., Pang L., Zhang M., Qu Y., Laster K.V., Dong Z. (2023). Cdc2-like kinases: structure, biological function, and therapeutic targets for diseases. Signal Transduction Targeted Ther..

[3] Nayler O., Stamm S., Ullrich A. (1997). Characterization and comparison of four serine-and arginine-rich (SR) protein kinases. Biochem. J..

[4] Yun B., Farkas R., Lee K., Rabinow L. (1994). The Doa locus encodes a member of a new protein kinase family and is essential for eye and embryonic development in Drosophila melanogaster. Genes Dev..

[5] D’Souza S.A., Rajendran L., Bagg R., Barbier L., van Pel D.M., Moshiri H. (2016). The MADD-3 LAMMER kinase interacts with a p38 MAP kinase pathway to regulate the display of the EVA-1 guidance receptor in Caenorhabditis elegans. PLoS Genet..

[6] Haltenhof T., Kotte A., De Bortoli F., Schiefer S., Meinke S., Emmerichs A.-K. (2020). A conserved kinase-based body-temperature sensor globally controls alternative splicing and gene expression. Mol. Cell.

[7] Virgirinia R.P., Nakamura M., Takebayashi-Suzuki K., Fatchiyah F., Suzuki A. (2021). The dual-specificity protein kinase Clk3 is essential for Xenopus. Neural Dev. Biochem. Biophysical Res. Commun..

[8] Virgirinia R.P., Jahan N., Okada M., Takebayashi-Suzuki K., Yoshida H., Nakamura M. (2019). Cdc2-like kinase 2 (Clk2) promotes early neural development in Xenopus embryos development. Growth Differ..

[9] Bender J., Fink G.R. (1994). AFC1, a LAMMER kinase from Arabidopsis thaliana, activates STE12-dependent processes in yeast. Proc. Natl. Acad. Sci. U. S. A..

[10] Lin J., Shi J., Zhang Z., Zhong B., Zhu Z. (2022). Plant AFC2 kinase desensitizes thermomorphogenesis through modulation of alternative splicing. Iscience.

[11] Kim K.-H., Cho Y.-M., Kang W.-H., Kim J.-H., Byun K.-H., Park Y.-D. (2001). Negative regulation of filamentous growth and flocculation by Lkh1, a fission yeast LAMMER kinase homolog. Biochem. Biophysical Res. Commun..

[12] Lim J.-Y., Park H.-M. (2019). The dual-specificity LAMMER kinase affects stress-response and morphological plasticity in fungi. Front. Cell Infect. Microbiol..

[13] Yamaguchi A., Iwatani M., Ogawa M., Kitano H., Matsuyama M. (2013). In vitro characterization of the RS motif in N-terminal head domain of goldfish germinal vesicle lamin B3 necessary for phosphorylation of the p34cdc2 target serine by SRPK1. FEBS Open Bio.

[14] Duan L., Xiao W., Xia F., Liu H., Xiao J., Li X. (2016). Two different transcripts of a LAMMER kinase gene play opposite roles in disease resistance. Plant Physiol..

[15] van Hooff J.J., Tromer E., van Dam T.J., Kops G.J., Snel B. (2019). Inferring the evolutionary history of your favorite protein: a guide for molecular biologists. BioEssays.

[16] Blackie A.C., Foley D.J. (2022). Exploring the roles of the Cdc2-like kinases in cancers. Bioorg Med Chem.

[17] Lee J.Y., Yun J.-S., Kim W.-K., Chun H.-S., Jin H., Cho S. (2019). Structural basis for the selective inhibition of Cdc2-like kinases by CX-4945. Biomed. Res. Int..

[18] Du C., McGuffin M.E., Dauwalder B., Rabinow L., Mattox W. (1998). Protein phosphorylation plays an essential role in the regulation of alternative splicing and sex determination in Drosophila. Mol. Cell.

[19] Golovkin M., Reddy A.S. (1999). An SC35-like protein and a novel serine/arginine-rich protein interact with Arabidopsis U1-70K protein. J. Biol. Chem..

[20] Wright C.J., Smith C.W., Jiggins C.D. (2022). Alternative splicing as a source of phenotypic diversity. Nat. Rev. Genet..

[21] Rhine C.L., Cygan K.J., Soemedi R., Maguire S., Murray M.F., Monaghan S.F. (2018). Hereditary cancer genes are highly susceptible to splicing mutations. PLoS Genet..

[22] ElHady A.K., El-Gamil D.S., Abadi A.H., Abdel-Halim M., Engel M. (2023). An overview of cdc2-like kinase 1 (Clk1) inhibitors and their therapeutic indications. Med. Res. Rev..

[23] Haltenhof T. (2019).

[24] Dimos-Röhl B., Ostwaldt F., Bäsmann J., Hausmann P., Kreisz P., Krischke M. (2024). AFC kinases function as thermosensors that regulate warm temperature-responsive growth in Arabidopsis. bioRxiv.

[25] Preußner M., Heyd F. (2018). Temperature-controlled rhythmic gene expression in endothermic mammals: all diurnal rhythms are equal, but some are circadian. BioEssays.

[26] Meyer C., Scalzitti N., Jeannin-Girardon A., Collet P., Poch O., Thompson J.D. (2020). Understanding the causes of errors in eukaryotic protein-coding gene prediction: a case study of primate proteomes. BMC Bioinformatics.

[27] Ko B.J., Lee C., Kim J., Rhie A., Yoo D.A., Howe K. (2022). Widespread false gene gains caused by duplication errors in genome assemblies. Genome Biol..

[28] Bagheri H., Severin A.J., Rajan H. (2020). Detecting and correcting misclassified sequences in the large-scale public databases. Bioinformatics.

[29] Deutekom E.S., Vosseberg J., Van Dam T.J., Snel B. (2019). Measuring the impact of gene prediction on gene loss estimates in Eukaryotes by quantifying falsely inferred absences. PLoS Comput. Biol..

[30] Chen Q., Zobel J., Verspoor K. (2017). Duplicates, redundancies and inconsistencies in the primary nucleotide databases: a descriptive study. Database (Oxford).

[31] Nagy A., Hegyi H., Farkas K., Tordai H., Kozma E., Bányai L. (2008). Identification and correction of abnormal, incomplete and mispredicted proteins in public databases. BMC Bioinformatics.

[32] Poirot O., Suhre K., Abergel C., O'Toole E., Notredame C. (2004). 3DCoffee@ igs: a web server for combining sequences and structures into a multiple sequence alignment. Nucleic Acids Res..

[33] Bowman J.P., Nichols D.S. (2005). Novel members of the family Flavobacteriaceae from Antarctic maritime habitats including Subsaximicrobium wynnwilliamsii gen. nov., sp. nov., Subsaximicrobium saxinquilinus sp. nov., Subsaxibacter broadyi gen. nov., sp. nov., Lacinutrix copepodicola gen. nov., sp. nov., and novel species of the genera Bizionia, Gelidibacter and Gillisia. Int. J. Syst. Evol. Microbiol..

[34] Hartman H., Fedorov A. (2002). The origin of the eukaryotic cell: a genomic investigation. Proc. Natl. Acad. Sci..

[35] van Wijk L.M., Snel B. (2020). The first eukaryotic kinome tree illuminates the dynamic history of present-day kinases. BioRxiv.

[36] Karnkowska A., Treitli S.C., Brzoň O., Novák L., Vacek V., Soukal P. (2019). The oxymonad genome displays canonical eukaryotic complexity in the absence of a mitochondrion. Mol. Biol. Evol..

[37] Fritz-Laylin L.K., Prochnik S.E., Ginger M.L., Dacks J.B., Carpenter M.L., Field M.C. (2010). The genome of Naegleria gruberi illuminates early eukaryotic versatility. Cell.

[38] Ishii M., Akiyoshi B. (2020). Characterization of unconventional kinetochore kinases KKT10 and KKT19 in Trypanosoma brucei. J. Cell Sci..

[39] Saldivia M., Fang E., Ma X., Myburgh E., Carnielli J.B., Bower-Lepts C. (2020). Targeting the trypanosome kinetochore with CLK1 protein kinase inhibitors. Nat. Microbiol..

[40] Saldivia M., Wollman A.J., Carnielli J.B., Jones N.G., Leake M.C., Bower-Lepts C. (2021). A CLK1-KKT2 signaling pathway regulating kinetochore assembly in Trypanosoma brucei. mBio.

[41] Geoghegan V., Carnielli J.B., Jones N.G., Saldivia M., Antoniou S., Hughes C. (2022). CLK1/CLK2-driven signalling at the Leishmania kinetochore is captured by spatially referenced proximity phosphoproteomics. Commun. Biol..

[42] Read B.A., Kegel J., Klute M.J., Kuo A., Lefebvre S.C., Maumus F. (2013). Pan genome of the phytoplankton Emiliania underpins its global distribution. Nature.

[43] Qiu H., Rossoni A.W., Weber A.P., Yoon H.S., Bhattacharya D. (2018). Unexpected conservation of the RNA splicing apparatus in the highly streamlined genome of Galdieria sulphuraria. BMC Evol. Biol..

[44] Curtis B.A., Tanifuji G., Burki F., Gruber A., Irimia M., Maruyama S. (2012). Algal genomes reveal evolutionary mosaicism and the fate of nucleomorphs. Nature.

[45] Gould S.B., Sommer M.S., Kroth P.G., Gile G.H., Keeling P.J., Maier U.-G. (2006). Nucleus-to-nucleus gene transfer and protein retargeting into a remnant cytoplasm of cryptophytes and diatoms. Mol. Biol. Evol..

[46] Sebé-Pedrós A., Irimia M., Del Campo J., Parra-Acero H., Russ C., Nusbaum C. (2013). Regulated aggregative multicellularity in a close unicellular relative of metazoa. eLife.

[47] Suga H., Chen Z., De Mendoza A., Sebé-Pedrós A., Brown M.W., Kramer E. (2013). The Capsaspora genome reveals a complex unicellular prehistory of animals. Nat. Commun..

[48] Dhabalia Ashok A., de Vries S., Darienko T., Irisarri I., andde Vries J. (2024). Evolutionary assembly of the plant terrestrialization toolkit from protein domains. Proc. B.

[49] Corradi N. (2015). Microsporidia: eukaryotic intracellular parasites shaped by gene loss and horizontal gene transfers. Annu. Rev. Microbiol..

[50] von der Dunk S.H.A., Snel B. (2020). Recurrent sequence evolution after independent gene duplication. BMC Evol. Biol..

[51] Lynch M., Force A. (2000). The probability of duplicate gene preservation by subfunctionalization. Genetics.

[52] Padmanabha R., Gehrung S., Snyder M. (1991). The KNS1 gene of Saccharomyces cerevisiae encodes a nonessential protein kinase homologue that is distantly related to members of the CDC28/cdc2 gene family. Mol. Gen. Genet..

[53] Choi Y.K., Kang E.-H., Park H.-M. (2014). Role of LAMMER kinase in cell wall biogenesis during vegetative growth of Aspergillus nidulans. Mycobiology.

[54] de Sena-Tomás C., Sutherland J.H., Milisavljevic M., Nikolic D.B., Pérez-Martín J., Kojic M. (2015). LAMMER kinase contributes to genome stability in Ustilago maydis. DNA Repair.

[55] Lim J.-Y., Park Y.-H., Pyon Y.-H., Yang J.-M., Yoon J.-Y., Park S.J. (2020). The LAMMER kinase is involved in morphogenesis and response to cell wall-and DNA-damaging stresses in Candida albicans. Med. Mycol..

[56] Lim J.-Y., Kim Y.J., Woo S.A., Jeong J.W., Lee Y.-R., Kim C.-H. (2021). The LAMMER kinase, LkhA, affects Aspergillus fumigatus pathogenicity by modulating reproduction and biosynthesis of cell wall PAMPs. Front. Cell Infect. Microbiol..

[57] Li L., Zhu X.-M., Wu J.-Q., Cao N., Bao J.-D., Liu X.-H. (2022). The LAMMER kinase MoKns1 regulates growth, conidiation and pathogenicity in Magnaporthe oryzae. Int. J. Mol. Sci..

[58] Smith J.J., Keinath M.C. (2015). The sea lamprey meiotic map improves resolution of ancient vertebrate genome duplications. Genome Res..

[59] Irisarri I., Mauro D.S., Abascal F., Ohler A., Vences M., Zardoya R. (2012). The origin of modern frogs (Neobatrachia) was accompanied by acceleration in mitochondrial and nuclear substitution rates. BMC Genomics.

[60] Wang K., Wang J., Zhu C., Yang L., Ren Y., Ruan J. (2021). African lungfish genome sheds light on the vertebrate water-to-land transition. Cell.

[61] Biscotti M.A., Adolfi M.C., Barucca M., Forconi M., Pallavicini A., Gerdol M. (2018). A comparative view on sex differentiation and gametogenesis genes in lungfish and coelacanths. Genome Biol. Evol..

[62] Otake S., Saito S., Lin X., Saito C.T., Kohno S., Takagi W. (2025). Functional characterizations of thermosensitive TRPV channels from Holocephalan elephant shark, Callorhinchus milii, illuminate the ancestral thermosensory system in vertebrates. J. Exp. Biol..

[63] Glasauer S.M., Neuhauss S.C. (2014). Whole-genome duplication in teleost fishes and its evolutionary consequences. Mol. Genet. Genomics.

[64] Davesne D., Friedman M., Schmitt A.D., Fernandez V., Carnevale G., Ahlberg P.E. (2021). Fossilized cell structures identify an ancient origin for the teleost whole-genome duplication. Proc. Natl. Acad. Sci. U. S. A..

[65] Busch A., Hertel K.J. (2012). Evolution of SR protein and hnRNP splicing regulatory factors Wiley Interdisciplinary Reviews. RNA.

[66] Keren H., Lev-Maor G., Ast G. (2010). Alternative splicing and evolution: diversification, exon definition and function. Nat. Rev. Genet..

[67] Barbosa-Morais N.L., Carmo-Fonseca M., Aparício S. (2006). Systematic genome-wide annotation of spliceosomal proteins reveals differential gene family expansion. Genome Res..

[68] Albalat R., Cañestro C. (2016). Evolution by gene loss. Nat. Rev. Genet..

[69] Yun B., Lee K., Farkaš R., Hitte C., Rabinow L. (2000). The LAMMER protein kinase encoded by the Doa locus of Drosophila is required in both somatic and germline cells and is expressed as both nuclear and cytoplasmic isoforms throughout development. Genetics.

[70] Hatting M., Rines A.K., Luo C., Tabata M., Sharabi K., Hall J.A. (2017). Adipose tissue CLK2 promotes energy expenditure during high-fat diet intermittent fasting. Cell Metab..

[71] Artarini A., Meyer M., Shin Y.J., Huber K., Hilz N., Bracher F. (2019). Regulation of influenza A virus mRNA splicing by CLK1. Antiviral Res..

[72] Groza T., Gomez F.L., Mashhadi H.H., Muñoz-Fuentes V., Gunes O., Wilson R. (2023). The International Mouse Phenotyping Consortium: comprehensive knockout phenotyping underpinning the study of human disease. Nucleic Acids Res..

[73] Wang Z., Gao X., Li Q., Zhu H., Zhao X., Garcia-Barrio M. (2021). Inhibition of a novel CLK1-THRAP3-PPARγ axis improves insulin sensitivity. Front. Physiol..

[74] Rancati G., Moffat J., Typas A., Pavelka N. (2018). Emerging and evolving concepts in gene essentiality. Nat. Rev. Genet..

[75] Acevedo-Rocha C.G., Fang G., Schmidt M., Ussery D.W., Danchin A. (2013). From essential to persistent genes: a functional approach to constructing synthetic life. Trends Genet..

[76] Fang G., Rocha E., Danchin A. (2005). How essential are nonessential genes?. Mol. Biol. Evol..

[77] Stumpf M.P., Thorne T., De Silva E., Stewart R., An H.J., Lappe M. (2008). Estimating the size of the human interactome. Proc. Natl. Acad. Sci. U. S. A..

[78] Hu Y., Flockhart I., Vinayagam A., Bergwitz C., Berger B., Perrimon N. (2011). An integrative approach to ortholog prediction for disease-focused and other functional studies. BMC Bioinformatics.

[79] Aleksander S.A., Balhoff J., Carbon S., Cherry J.M., Drabkin H.J., Ebert D. (2023). The gene ontology knowledgebase in 2023. Genetics.

[80] Ashburner M., Ball C.A., Blake J.A., Botstein D., Butler H., Cherry J.M. (2000). Gene ontology: tool for the unification of. Biol. Nat. Genet..

[81] Nikolakaki E., Du C., Lai J., Giannakouros T., Cantley L., Rabinow L. (2002). Phosphorylation by LAMMER protein kinases: determination of a consensus site, identification of in vitro substrates, and implications for substrate preferences. Biochemistry.

[82] Lipp J.J., Marvin M.C., Shokat K.M., Guthrie C. (2015). SR protein kinases promote splicing of nonconsensus introns. Nat. Struct. Mol. Biol..

[83] Prasad J., Manley J.L. (2003). Regulation and substrate specificity of the SR protein kinase Clk/Sty. Mol. Cell Biol..

[84] Rabinow L., Samson M.-L. (2010). The role of the Drosophila LAMMER protein kinase DOA in somatic sex determination. J. Genet..

[85] Savaldi-Goldstein S., Aviv D., Davydov O., Fluhr R. (2003). Alternative splicing modulation by a LAMMER kinase impinges on developmental and transcriptome expression. Plant Cell.

[86] Ast G. (2004). How did alternative splicing evolve?. Nat. Rev. Genet..

[87] Vosseberg J., Stolker D., von der Dunk S.H., Snel B. (2023). Integrating phylogenetics with intron positions illuminates the origin of the complex spliceosome. Mol. Biol. Evol..

[88] Schreiber K., Csaba G., Haslbeck M., Zimmer R. (2015). Alternative splicing in next generation sequencing data of Saccharomyces cerevisiae. PLoS One.

[89] Fang S., Hou X., Qiu K., He R., Feng X., Liang X. (2020). The occurrence and function of alternative splicing in fungi. Fungal Biol. Rev..

[90] Marshall N.M. (2012).

[91] Montañés J.C., Huertas M., Moro S.G., Blevins W.R., Carmona M., Ayté J. (2022). Native RNA sequencing in fission yeast reveals frequent alternative splicing isoforms. Genome Res..

[92] Tang Z., Luca M., Portillio J., Ngo B., Chang C., Wen T. (2011). LAMMER kinase Kic1 is involved in pre-mRNA processing. Exp. Cell Res..

[93] Jiang K., Patel N.A., Watson J.E., Apostolatos H., Kleiman E., Hanson O. (2009). Akt2 regulation of Cdc2-like kinases (Clk/Sty), serine/arginine-rich (SR) protein phosphorylation, and insulin-induced alternative splicing of PKCβII messenger ribonucleic acid. Endocrinology.

[94] Petsalaki E., Zachos G. (2016). Clks 1, 2 and 4 prevent chromatin breakage by regulating the Aurora B-dependent abscission checkpoint. Nat. Commun..

[95] Morris J.Z., Navarro C., Lehmann R. (2003). Identification and analysis of mutations in bob, Doa and eight new genes required for oocyte specification and development in Drosophila melanogaster. Genetics.

[96] Takeuchi M., Yanagida M. (1993). A mitotic role for a novel fission yeast protein kinase dsk1 with cell cycle stage dependent phosphorylation and localization. Mol. Biol. Cell.

[97] Tang Z., Tsurumi A., Alaei S., Wilson C., Chiu C., Oya J. (2007). Dsk1p kinase phosphorylates SR proteins and regulates their cellular localization in fission yeast. Biochem. J..

[98] Abramson J., Adler J., Dunger J., Evans R., Green T., Pritzel A. (2024). Accurate structure prediction of biomolecular interactions with AlphaFold 3. Nature.

[99] Bullock A.N., Das S., Debreczeni J.É., Rellos P., Fedorov O., Niesen F.H. (2009). Kinase domain insertions define distinct roles of CLK kinases in SR protein phosphorylation. Structure.

[100] Kumar S., Suleski M., Craig J.M., Kasprowicz A.E., Sanderford M., Li M. (2022). TimeTree 5: an expanded resource for species Divergence times. Mol. Biol. Evol..

[101] Hudson A.J., Stark M.R., Fast N.M., Russell A.G., Rader S.D. (2015). Splicing diversity revealed by reduced spliceosomes in C. merolae and other organisms. RNA Biol..

[102] Schwartz S., Silva J., Burstein D., Pupko T., Eyras E., Ast G. (2008). Large-scale comparative analysis of splicing signals and their corresponding splicing factors in eukaryotes. Genome Res..

[103] Aubol B.E., Adams J.A. (2014). Recruiting a silent partner for activation of the protein kinase SRPK1. Biochemistry.

[104] Aubol B.E., Plocinik R.M., McGlone M.L., Adams J.A. (2012). Nucleotide release sequences in the protein kinase SRPK1 accelerate substrate phosphorylation. Biochemistry.

[105] Kpebe A., Rabinow L. (2008). Alternative promoter usage generates multiple evolutionarily conserved isoforms of Drosophila DOA kinase. Genesis.

[106] Ishida T., Kinoshita K. (2007). PrDOS: prediction of disordered protein regions from amino acid sequence. Nucleic Acids Res..

[107] Zarin T., Strome B., Nguyen Ba A.N., Alberti S., Forman-Kay J.D., Moses A.M. (2019). Proteome-wide signatures of function in highly diverged intrinsically disordered regions. eLife.

[108] Keshwani M.M., Hailey K.L., Aubol B.E., Fattet L., McGlone M.L., Jennings P.A. (2015). Nuclear protein kinase CLK1 uses a non-traditional docking mechanism to select physiological substrates. Biochem. J..

[109] Aubol B.E., Plocinik R.M., Keshwani M.M., McGlone M.L., Hagopian J.C., Ghosh G. (2014). N-terminus of the protein kinase CLK1 induces SR protein hyperphosphorylation. Biochem. J..

[110] Lee K., Du C., Horn M., Rabinow L. (1996). Activity and autophosphorylation of LAMMER protein kinases. J. Biol. Chem..

[111] Yu E.-Y., Lee J.-H., Kang W.-H., Park Y.-H., Kim L., Park H.-M. (2013). Fission yeast LAMMER kinase Lkh1 regulates the cell cycle by phosphorylating the CDK-inhibitor Rum1. Biochem. Biophysical Res. Commun..

[112] Lee J., Moir R.D., McIntosh K.B., Willis I.M. (2012). TOR signaling regulates ribosome and tRNA synthesis via LAMMER/Clk and GSK-3 family kinases. Mol. Cell.

[113] Rodgers J.T., Haas W., Gygi S.P., Puigserver P. (2010). Cdc2-like kinase 2 is an insulin-regulated suppressor of hepatic gluconeogenesis. Cell Metab..

[114] Farkaš R., Kovacikova M., Liszekova D., Beno M., Danis P., Rabinow L.J. (2009). Exploring some of the physico-chemical properties of the LAMMER protein kinase DOA of Drosophila. Fly (Austin).

[115] Nolen B., Taylor S., Ghosh G. (2004). Regulation of protein kinases: controlling activity through activation segment conformation. Mol. Cell.

[116] Elias M., Wieczorek G., Rosenne S., Tawfik D.S. (2014). The universality of enzymatic rate–temperature dependency. Trends Biochem. Sci..

[117] Worley A.C., Raper K.B., Hohl M. (1979). Fonticula alba: a new cellular slime mold. Acrasiomycetes) Mycologia.

[118] Neumann A., Meinke S., Goldammer G., Strauch M., Schubert D., Timmermann B. (2020). Alternative splicing coupled mRNA decay shapes the temperature-dependent transcriptome. EMBO Rep..

[119] Buhr E.D., Yoo S.-H., Takahashi J.S. (2010). Temperature as a universal resetting cue for mammalian circadian oscillators. Science.

[120] Goldammer G., Neumann A., Strauch M., Müller-McNicoll M., Heyd F., Preußner M. (2018). Characterization of cis-acting elements that control oscillating alternative splicing. RNA Biol..

[121] Preußner M., Goldammer G., Neumann A., Haltenhof T., Rautenstrauch P., Müller-McNicoll M. (2017). Body temperature cycles control rhythmic alternative splicing in mammals. Mol. Cell.

[122] Morf J., Rey G., Schneider K., Stratmann M., Fujita J., Naef F. (2012). Cold-inducible RNA-binding protein modulates circadian gene expression posttranscriptionally. Science.

[123] Gotic I., Omidi S., Fleury-Olela F., Molina N., Naef F., Schibler U. (2016). Temperature regulates splicing efficiency of the cold-inducible RNA-binding protein gene Cirbp. Genes Dev..

[124] Li B.J., Zhu Z.X., Qin H., Meng Z.N., Lin H.R., Xia J.H. (2020). Genome-wide characterization of alternative splicing events and their responses to cold stress in tilapia. Front. Genet..

[125] Colot H.V., Loros J.J., Dunlap J.C. (2005). Temperature-modulated alternative splicing and promoter use in the circadian clock gene frequency. Mol. Biol. Cell.

[126] Diernfellner A., Colot H.V., Dintsis O., Loros J.J., Dunlap J.C., Brunner M. (2007). Long and short isoforms of Neurospora clock protein FRQ support temperature-compensated circadian rhythms. FEBS Lett..

[127] Shiina T., Shimizu Y. (2020). Temperature-dependent alternative splicing of precursor mRNAs and its biological significance: a review focused on post-transcriptional regulation of a cold shock protein gene in hibernating mammals. Int. J. Mol. Sci..

[128] Majercak J., Sidote D., Hardin P.E., Edery I. (1999). How a circadian clock adapts to seasonal decreases in temperature and day length. Neuron.

[129] Martin Anduaga A., Evantal N., Patop I.L., Bartok O., Weiss R., Kadener S. (2019). Thermosensitive alternative splicing senses and mediates temperature adaptation in Drosophila. eLife.

[130] Preußner M., Wilhelmi I., Schultz A.-S., Finkernagel F., Michel M., Möröy T. (2014). Rhythmic U2af26 alternative splicing controls PERIOD1 stability and the circadian clock in mice. Mol. Cell.

[131] Czerwinski M., Natarajan A., Barske L., Looger L.L., Capel B. (2016). A timecourse analysis of systemic and gonadal effects of temperature on sexual development of the red-eared slider turtle Trachemys scripta elegans. Dev. Biol..

[132] Deveson I.W., Holleley C.E., Blackburn J., Marshall Graves J.A., Mattick J.S., Waters P.D. (2017). Differential intron retention in Jumonji chromatin modifier genes is implicated in reptile temperature-dependent sex determination. Sci. Adv..

[133] Rosembert M. (2017).

[134] Twyffels L., Gueydan C., Kruys V. (2011). Shuttling SR proteins: more than splicing factors. FEBS J..

[135] Nadeem S.G., Shafiq A., Hakim S.T., Anjum Y., Kazm S.U. (2013). Effect of growth media, pH and temperature on yeast to hyphal transition in Candida albicans. Open J. Med. Microbiol..

[136] Gallo M., Giovati L., Magliani W., Pertinhez T.A., Conti S., Ferrari E. (2022). Metabolic plasticity of Candida albicans in response to different environmental conditions. J. Fungi.

[137] Park Y.-H., Park H.-M. (2011). Temperature sensitivity of sigma background is suppressed by the disruption of ScKNS1 in Saccharomyces cerevisiae Korean. J. Microbiol..

[138] Katoh K., Standley D.M. (2013). MAFFT multiple sequence alignment software version 7: improvements in performance and usability. Mol. Biol. Evol..

[139] Tamura K., Stecher G., Kumar S. (2021). MEGA11: molecular evolutionary genetics analysis version 11. Mol. Biol. Evol..

[140] Kozlov A.M., Darriba D., Flouri T., Morel B., Stamatakis A. (2019). RAxML-NG: a fast, scalable and user-friendly tool for maximum likelihood phylogenetic inference. Bioinformatics.

[141] Morel B., Kozlov A.M., Stamatakis A., Szöllősi G.J. (2020). GeneRax: a tool for species-tree-aware maximum likelihood-based gene family tree inference under gene duplication, transfer, and loss. Mol. Biol. Evol..

[142] Letunic I., Bork P. (2021). Interactive tree of life (iTOL) v5: an online tool for phylogenetic tree display and annotation. Nucleic Acids Res..

[143] Oughtred R., Rust J., Chang C., Breitkreutz B.J., Stark C., Willems A. (2021). The BioGRID database: a comprehensive biomedical resource of curated protein, genetic, and chemical interactions. Protein Sci..

[144] Robert X., Gouet P. (2014). Deciphering key features in protein structures with the new ENDscript server. Nucleic Acids Res..

[145] Meng E.C., Goddard T.D., Pettersen E.F., Couch G.S., Pearson Z.J., Morris J.H. (2023). UCSF ChimeraX: tools for structure building and analysis. Protein Sci..

