# Supplementary material for: Phylogenetic and structural analyses reveal Cdc2-like kinases (CLKs) as ancient regulators of thermosensitive splicing
Source: J Biol Chem. 2025 Nov 26;302(1):110979. doi: 10.1016/j.jbc.2025.110979 (PMC12795684; doi:10.1016/j.jbc.2025.110979)
Supplement: Supporting Data Legends [file mmc1.pdf]

Supporting Figure 1. pLDDT values for CLK structures.

AlphaFold-3 structural predictions are shown with colours to match their pLDDT confidence scores from (A) Figure 5 and (B) Figure 8. Colour legend below to match matches that used by Alphafold.

Supporting Figure 2. Disorder of CLKs across diverse eukaryotes.

The disorder probability of CLK sequences from diverse eukaryotes was scored for each residue using PrDOS. Residues are scored as disordered when above 0.5 (red line). The start of the kinase domain is marked by the orange arrow, and the end is marked by the green arrow.

Supporting Figure 3. Distribution of serine and arginine residues in the N-termini of CLK proteins.

N-terminal sequences of CLK proteins from diverse eukaryotic species were analysed for serine (S) and arginine (R) content. The percentage of S and R residues was calculated by summing their total counts and dividing by the total number of amino acids. Similarly, the frequency of SR and RS dipeptide motifs was determined by combining the number of "SR" and "RS" motifs and dividing by the total sequence length.

Supporting Figure 3. The activation segment in CLKs across eukaryotes.

A phylogenetic tree illustrating the relationships among CLK proteins. Adjacent to each CLK are aligned activation segment sequences followed by taxonomy. Below is a graph displaying the frequency of amino acids matching the consensus sequence.

Supporting Table 1. Complete search results for eukaryotic CLKs.

HMM profiles were generated for CLKs and searched in the NCBI RefSeq eukaryotic protein database (release #228). "TOTAL": total search results, "TREE": selections for the phylogenetic analysis, "Genes": no grouping, "Species": grouped by species.

Supporting Table 2. Interolog mapping of CLK binding proteins.

List of CLK interacting proteins and corresponding databases retrieved from. Homologous pairs identified using DIOPT ortholog finder and functional information retrieved from Gene Ontology.

Supporting Table 3. List of proteins used for Figure 5.

CLK homologs and their corresponding sequences used to generate AlphaFold3 structures.
