# Supplementary material for: Phylogenetic and structural analyses reveal Cdc2-like kinases (CLKs) as ancient regulators of thermosensitive splicing
Source: J Biol Chem. 2025 Nov 26;302(1):110979. doi: 10.1016/j.jbc.2025.110979 (PMC12795684; doi:10.1016/j.jbc.2025.110979)
Supplement: Supporting Figures [file mmc2.pdf]

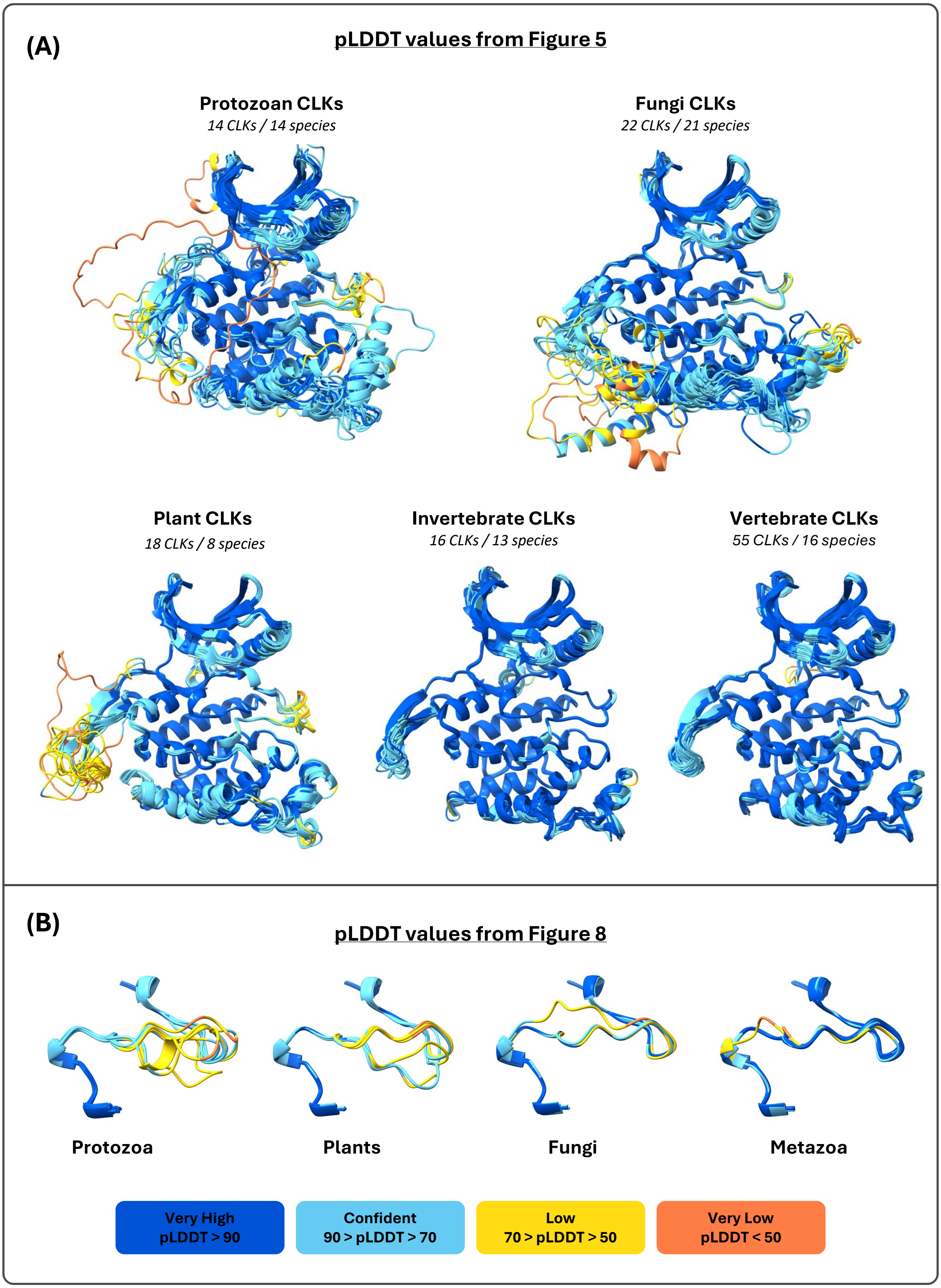

Supporting Figure 1

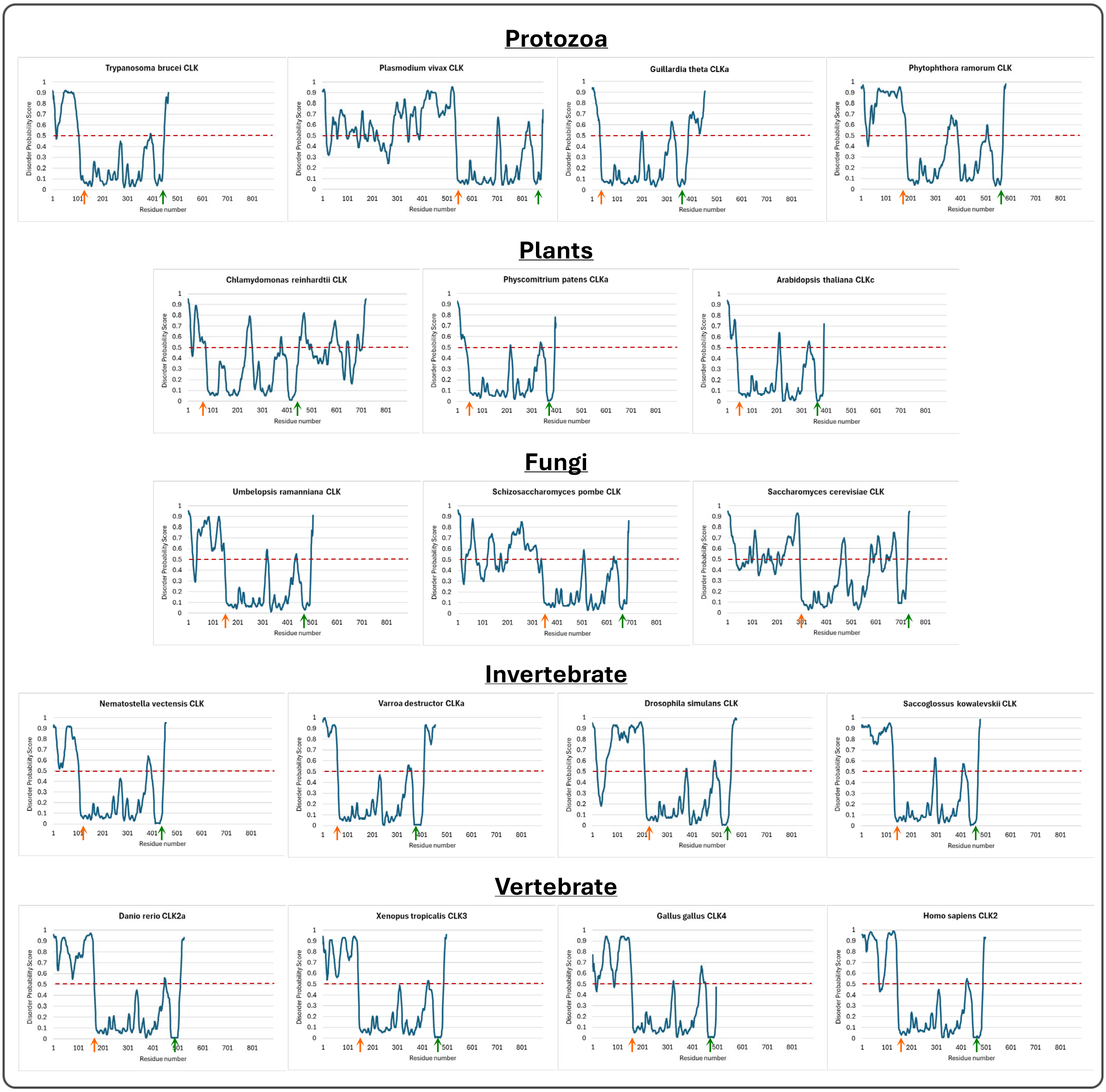

Supporting Figure 2

	Species CLK	N-terminal sequence	Percentage S & R combined	Percentage SR/RS motifs
Protozoa	**Trypanosoma brucei CLK**	MDSISSGLTHRAGLQQYPSGLVNVHVNNNPKINNGACSNMTPRESTNSARRSGSKRDRETADTADDGRNKNNQSITGTTATTTSTAKHSEMQPPPKKKKVTYALPNQSREEG	19%	4%
	**Plasmodium vivax CLK**	MIQENYKDGSEKYSSKYREYNYYRSYISDIKGKYMGFGNMQRDSPNGRYFNDPRRNNNYYYRNREGNLHDGAYGNSHRNSHRNSYRNSYKNLHKNLHKNLHKNLHKNSYQNSYQNSYQNGYHDSHNHVYNSGFGNAYGNPYGSGFKLLKGKRIRSFNSTSNSSRKGKLFKKFKNYDVDENMIRAYKNGRKRSYSSMREINDREQRHHKVYLESKYHHEEHQNNYLKGFDFTRKRNYYSYKKKLYNSDRRNYDTHFGQGNIFYNNEYYLFNDGATLKKKKMYYGNKNSFRTNSRSYNGHSDRMNNKRKDSNRNGKYKATAVHQKETGSLGGGPSATSATSAATAVRICSYKDKDDKSNKSFKLKNTDTTRDDGTGKSDVRDEIMGSRRNLVVCSDKEKGSQQSLRKYTRGVRKRRKAPSSYSGTRSGRRSRRRSRRRSRRRNAQRRRRKRDSLSSNEETGTSALSRRGSSYSSVENKKKNHADRYSSESYRYTPSDESTDDISSRKKYNHKRNRTKFSDMEDNNNRKKKKKKKENYDSDDEIV	25%	6%
	**Phytophthora ramorum CLK**	MERRRAGDSDGRRRRSRSFSPVSSRLRYQLRRRHPRPPPVPSSSHRRRRRLTKDAPVDGKRSRSRSRSHRGSRSSRRRTDSRAPRSRSRSRSGSRKRRHSGSSGSHRHHRRYSGRKTRTSRTDSQTRGRRNSPENDRGGRRRVSRGKNKQRHSRRRDSHHDREPLPPPPAAAQKDGSDDSASHDDTVGS	49%	28%
	**Guillardia theta CLKa**	MRVGGEKRPLRQDLQDYDPRQRRRTDEVKYPDHQWDDKDG	18%	0%
Plants	**Chlamydomonas reinhardtii CLK**	MPADEVASSSLRPGQGPLTVKRLYQSGSNESTEVSSSRKRARLHGEPIVRPGAPLPISLSAFRVPDKLSPPLREDDKDG	25%	3%
	**Physcomitrium patens CLKa**	MQSHQVAVNSGQYEQLGLGRGVPPAAIGNSPDFYNGYARVASPAWREDDKDG	13%	0%
	**Arabidopsis thaliana CLKc "AFC3"**	MDKERVRKRPRMTWDEAPAEPEAKRAVIKGHGSDGRILSPPLRDDDRDG	20%	0%
Fungi	**Umbelopsis ramanniana CLK**	MTTVATRRRKRSPNTDWKADFYRNGYPTEVIVIEDSPTPAPDQHTNSTSNSNNPQLNHASAYTTPPAASTRSKRPRRKVTSTYQPQLAPTSQISYPVLTPVSSLVGNFTQPAAKRRRKENGDAAGYSEASHRQDHVYRPTATTETAAANYDDKDG	19%	3%
	**Saccharomyces cerevisiae CLK "KNS1"**	MSQNIQIGTRKRSRANMNNSTTTGPANNTSSNKTFLDNFEETRTNKLLDEMFARQNSFLTDNLRNSLDLNQADNPLRPRQHQHQLFLDNENAIELDEEPRIINTTINNSNNNHNSSRVDEDADDDIIFIKEQPIQFSSPLILPSSSSINNNNNIVTSNNPGCGTAATSNSTYITTPKKFKKQRTISLPQLPLSKLSYQSNYFNVPDQTNAIVPRVTQTENELLHLTGSCAKTLEGNKAVNLTIAHSTSPFSNPPAQIASLPQSNLKKQIGSSLRKFTSNGSSESASSNKSNFKTDKDG	17%	2%
	**Schizosaccharomyces pombe CLK "LKH1"**	MHSLKRRRNHAPDWQDFYKNGVPQEVIVIEDSASPRLTPNLPPPFSVHQLQSFVPPQPPSSSSPSTTGTVAVPINGANAVYPSTNSVSLPQSYDPWLDANGVVPLPHDVASHPSYMVQSPTSYHACSNNQSPFPHSHHPPLHNPLPVSCQPVLRPPPVPQVPSHWYPVSLPSPNLPHQPISKPPVIPNLPKLQVHPNRLPHPIHNHPYSSPTSYPPPLCPATYCPSNPPQLAPATAIAPSSQSSQHKSVNYSVTPSSINNHTAVPLSPTLAVWLPMTQPTFQPPSANVYQPASNANQVITPVSISDYRPPKKRKRAAWPPYKKVDRVNVPVVHDTTAFDPSTFDDDDG	16%	0%
Invertebrate	**Nematostella vectensis CLK**	MRMPRSRRRRRSVCSRDTSPSTLYPRHYLDERLDRNCSPLEEVRFSRKHIRSRSRDRFRHHHYRERSRSSERSTHRRRHRHHNDVTDGYSIYTRSHAEERCYSTSRDDEDG	41%	27%
	**Varroa destructor CLKa**	MTHSSKSSSHRRRRSSSASYRRKRRCRERSRQFADHRAGSSKGDRSRSRSRSRSRSPLSRTPIQDDSDG	57%	43%
	**Drosophila simulans CLK "DOA"**	MQLPSPKDKHMPSGSVQQAKANFSWQSLTQLLSGLWQRLYLPRSPFLALPAPPLATPPANTTQRRAKKEMPRTRRLHHSRDRSSAGTRDKRRRHDTADHSPPLAEAPSPPRITNTHHTRSAAKRRRHELDAKKAQISKEPTFDDSISSRRRKERSKRSHRKSPAASRRQHKYRYRDETSHSSSRRRHRDRAKDERDSGRNNRQSQAKTAKPVIQDDADG	29%	8%
	**Saccoglossus kowalevskii CLK**	MPRSYHRYKSRSRSLRSSHHRHKRRRRSSSSGRRYEDKYYESPGSDYRRERSLSPERQIFRDNHYNDRSESGYYENYSRHYYHEEKDKYKSKDENYQSSYRKHKHKRRKHHSHSCSSTRSNKDQQRSPSVEDDEDG	38%	18%
Vertebrate	**Danio rerio CLK2a**	MPHSRRYPSSERASRSSYQDRYRERDRDRDRGRKPRHRRSPTYSSSSERERRARGHRQDTGYARSRSYDNRSSDRRPYDRHYGESYRRLDHSRERDRDQNRDHRTPSNYYSRDASPSYDFRRGRDRDREDSYRRKGSRRKHKRRRRRTRSYSASSSRSDSGTRALCVRDDEEG	47%	16%
	**Xenopus tropicalis CLK3**	MHRRKRYWSPKEGDIVQTWKRRRRSPSRERDGRRLHTHRISPRAHRFSPYRSCLRSWKRDTFFDRCKRTHSSEIRFSQGHSTSSHSKSYSYSTRSPARPHSRDRTKTKAEKQPHKISHKHSTRSGSYSSSKSQRSTKDSQNVENDKE	37%	11%
	**Gallus gallus CLK4**	MYLFEHRIPAYNVQQRKLEQMRHSKQSHCPEWDDRRSWDQRKHSSSRKRRKRSHSSGQESKHYKPNHVSESHYLDDRTINERDHHDRRYVEEYRNDFCEVYDHRHYHRDYEKSHHHHYSKSSGRSRKSSHKRKHKRHHCSSHQSHSKSHRRKRSRSVEDDEEG	31%	10%
	**Homo sapiens CLK2**	MPHPRRYHSSERGSRGSYREHYRSRKHKRRRSRSWSSSSDRTRRRRREDSYHVRSRSSYDDRSSDRRVYDRRYCGSYRRNDYSRDRGDAYYDTDYRHSYEYQRENSSYRSQRSSRRKHRRRRRRSRTFSRSSSQHSSRRAKSVEDDAEG	52%	26%

Supporting Figure 3

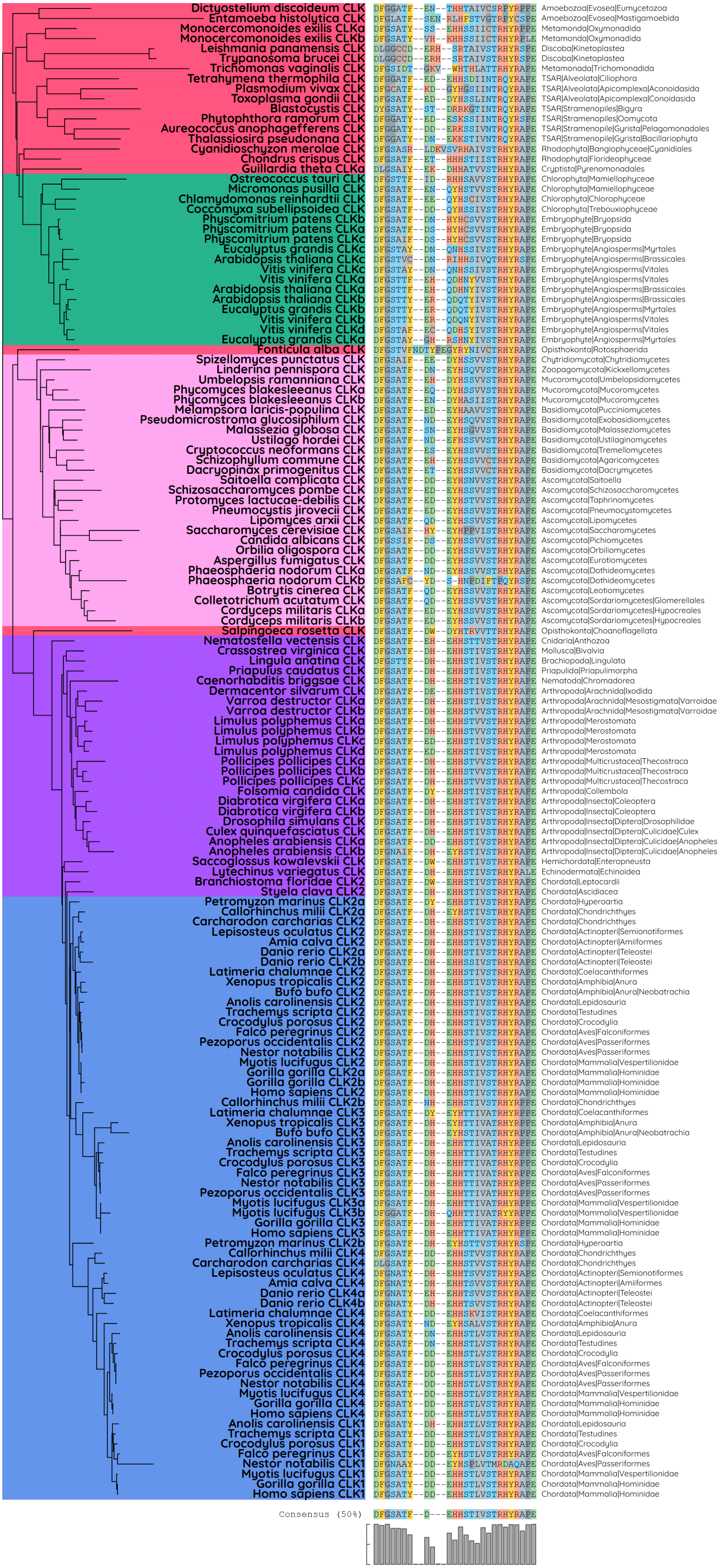

Supporting Figure 4
